# Contribution of S4 segments and S4-S5 linkers to the low-voltage activation properties of T-type Ca_V_3.3 channels

**DOI:** 10.1371/journal.pone.0193490

**Published:** 2018-02-23

**Authors:** Ana Laura Sanchez-Sandoval, Zazil Herrera Carrillo, Clara Estela Díaz Velásquez, Dulce María Delgadillo, Heriberto Manuel Rivera, Juan Carlos Gomora

**Affiliations:** 1 Departamento de Neuropatología Molecular, División de Neurociencias, Instituto de Fisiología Celular, Universidad Nacional Autónoma de México Mexico City, México; 2 Programa de Neurociencias, Facultad de Estudios Superiores Iztacala, Universidad Nacional Autónoma de México, Tlalnepantla de Baz, Estado de México, México; 3 Laboratorios Nacionales de Servicios Experimentales Centro de Investigación y de Estudios Avanzados del Instituto Politécnico Nacional, Mexico City, México; 4 Facultad de Medicina, Universidad Autónoma del Estado de Morelos Cuernavaca, Morelos, México; Indiana University School of Medicine, UNITED STATES

## Abstract

Voltage-gated calcium channels contain four highly conserved transmembrane helices known as S4 segments that exhibit a positively charged residue every third position, and play the role of voltage sensing. Nonetheless, the activation range between high-voltage (HVA) and low-voltage (LVA) activated calcium channels is around 30–40 mV apart, despite the high level of amino acid similarity within their S4 segments. To investigate the contribution of S4 voltage sensors for the low-voltage activation characteristics of Ca_V_3.3 channels we constructed chimeras by swapping S4 segments between this LVA channel and the HVA Ca_V_1.2 channel. The substitution of S4 segment of Domain II in Ca_V_3.3 by that of Ca_V_1.2 (chimera IIS4C) induced a ~35 mV shift in the voltage-dependence of activation towards positive potentials, showing an *I-V* curve that almost overlaps with that of Ca_V_1.2 channel. This HVA behavior induced by IIS4C chimera was accompanied by a 2-fold decrease in the voltage-dependence of channel gating. The IVS4 segment had also a strong effect in the voltage sensing of activation, while substitution of segments IS4 and IIIS4 moved the activation curve of Ca_V_3.3 to more negative potentials. Swapping of IIS4 voltage sensor influenced additional properties of this channel such as steady-state inactivation, current decay, and deactivation. Notably, Domain I voltage sensor played a major role in preventing Ca_V_3.3 channels to inactivate from closed states at extreme hyperpolarized potentials. Finally, site-directed mutagenesis in the Ca_V_3.3 channel revealed a partial contribution of the S4-S5 linker of Domain II to LVA behavior, with synergic effects observed in double and triple mutations. These findings indicate that IIS4 and, to a lesser degree IVS4, voltage sensors are crucial in determining the LVA properties of Ca_V_3.3 channels, although the accomplishment of this function involves the participation of other structural elements like S4-S5 linkers.

## Introduction

T-type calcium or Ca_V_3 channels are low-voltage activated (LVA) calcium channels that, together with high-voltage activated (HVA) calcium channels, are key elements in regulating calcium influx in most cells [1]. In particular, T-type calcium channels fulfill such function by activating close to the cells’ resting potential, which allows these channels to participate in several cell functions as neuronal burst firing [2–4], neurotransmitter and hormone release [5–8], proliferation and differentiation [9], and vasomotor function [10–12]. Therefore, T-type calcium channels are important pharmacological targets in pathophysiological processes like epilepsy [13–16], sleep disorders [17,18], hypertension [19–21] and cancer [22–25].

All voltage-gated calcium channels (LVA and HVA) are formed by a main pore-forming subunit (α1), which consists of four repeated domains (I to IV), each of them containing six transmembrane segments (S1 to S6). Segments S1 to S4 constitute the voltage sensor domain (VSD) with the S4 segment acting as the voltage sensor (characterized by several positively-charged residues, arginines or lysines); and the pore region is formed by segments S5, S6 and the membrane-associated loop between them [26]. It has been demonstrated that the movement of the S4 segment is responsible for the opening and closing of the voltage-gated channels [27,28]. However, to date there are just a few structure-function studies in Ca_V_3 channels focused to elucidate the molecular substrates responsible for the low-voltage activation characteristics of these channels. Two previous studies performed by the group of Wray and colleagues [29,30], using chimeras between the LVA (Ca_V_3.1) and HVA (Ca_V_1.2) calcium channels, have suggested that Domains I, III and IV are decisive for channel opening and each Domain, as a whole, strongly contributes to the difference in voltage dependence of activation between Ca_V_3.1 and Ca_V_1.2 channels. Nevertheless, this difference in voltage dependence was not observed when only the individual S4 voltage sensors in domains I, III and IV were swapped between the LVA and HVA channels. Moreover, the molecular substrate for determining the voltage dependence of activation of Domain I was found to be the pore region rather than the VSD. Unfortunately, these studies were focused on the voltage dependence of activation characteristic, whereas other LVA properties of the Ca_V_3.1 T-type calcium channel were not investigated.

Two additional studies about the role of individual charged residues in the S4 segments have been reported. Both studies found no significant role in the voltage sensing of activation for the outermost arginine (R1) in the IVS4 of both Ca_V_3.1 [31] and Ca_V_3.2 channels [32]. On the contrary, the second (R2) and third (R3) outermost arginines had a contribution in voltage-sensing activation, but they were not involved in the channel inactivation from the open state. However, the effects of these arginines on the voltage-dependence of inactivation were not evaluated. More recently, Morino et al. [33] reported a heterozygous mutation in 13 individuals from two different Japanese families diagnosed with spinocerebellar ataxia (SCA). The mutation was found to be located in S4 segment of repeat IV of Ca_V_3.1, introducing a histidine instead of the third outermost arginine (R3) of this S4 voltage sensor (Ca_V_3.1-R1715H). Contrasting with the absence of effect on voltage dependence of activation of the exact same mutation on Ca_V_3.2 reported by Lam [31], the mutant channel Ca_V_3.1-R1715H from the SCA individuals resulted in robust effects on the voltage dependence of activation and inactivation, by shifting the gating to more positive potentials [33]. Interestingly, when the same mutation was introduced into a different splice variant of the Ca_V_3.1 channel, the results showed only a discrete, although significant (4 mV), effect on the voltage sensing of activation toward more positive potentials. Such kind of contrasting results suggest that the gating of Ca_V_3 channels might be related to different interactions between S4 voltage sensors and other channel structures not canonically related to the voltage sensing in voltage-gate channels, even when the S4 segment sequences are quite similar among Ca_V_3 channels.

The third member of Ca_V_3 calcium channels subfamily is the Ca_V_3.3 channel [34], which is predominantly expressed in brain and in peripheral primary sensory neurons [35,36]. Recently it has been shown that Ca_V_3.3 channels trigger synaptic plasticity in reticular thalamic neurons, nRT [37,38]. In addition, Ca_V_3.3 channels dominate nRt rhythmic bursting and mediates a substantial fraction of spindle power in the NREM sleep EEG [39,40]. Structure-function studies like those mentioned above for Ca_V_3.1 and Ca_V_3.2, have not been reported for Ca_V_3.3 channels, with the exception of one from Zamponi’s group [41], which suggested a role of Domains I and IV in the control of voltage dependence, using chimeras between Ca_V_3.1 and Ca_V_3.3 channels. However, the role of individual S4 segments to the LVA properties of Ca_V_3.3 channels has not been evaluated. In the present study, we have investigated the contribution of the voltage sensors of each individual S4 segments for the low-voltage activated characteristics of the Ca_V_3.3 T-type calcium channels. Our strategy consisted in constructing single and double chimeras where the S4 voltage sensors of Ca_V_3.3 channels were substituted with the corresponding S4 segments of the HVA Ca_V_1.2 channel. Then, by using the whole-cell patch-clamp technique we systematically analyzed the biophysical properties of the constructs expressed in HEK-293 cells. Our results point out to a predominant role of the IIS4 voltage sensor in determining most of the low-voltage activation behavior of the Ca_V_3.3 channels with an additional contribution of IVS4. Furthermore, our evidences from site-directed mutagenesis suggest that the critical role of the voltage sensor of Domain II involves the participation of some residues from its own S4-S5 linker.

## Materials and methods

### Construction of calcium channel chimeras and mutants

**Fig 1:**
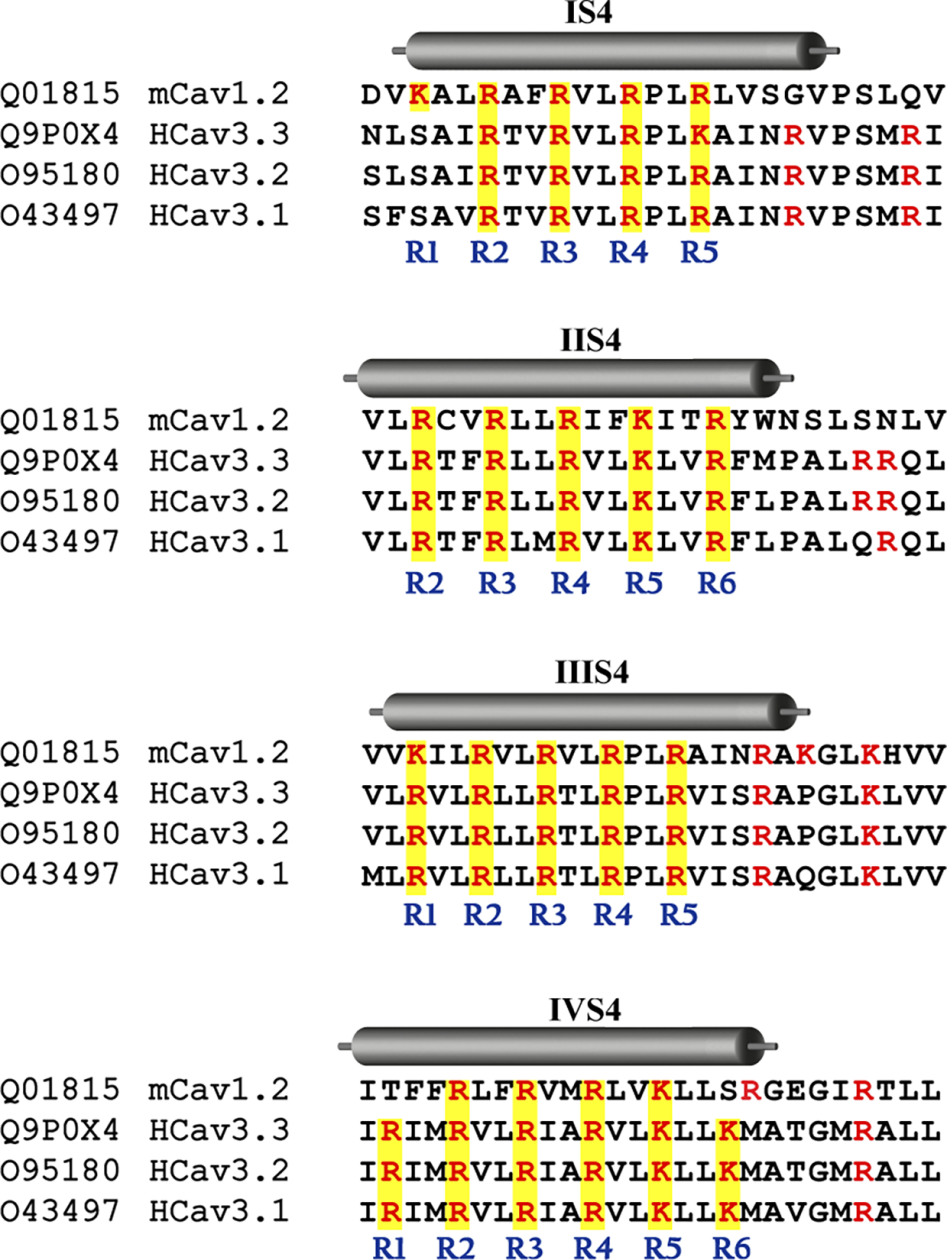
Sequence of the S4 segments of Ca_V_1.2 and Ca_V_3.3 α1 subunits. Amino acid sequence alignment of the S4 segments that were swapped between Ca_V_1.2 and Ca_V_3.3 channels. Positively charged residues (K, lysines and R, arginines) are colored in red, and those shaded yellow (labelled R1-R6) are the equivalent residues that are involved in the gating of Shaker K^+^ channel [27]. The cylinders depicting S4 segments are restricted to the structural data reported recently by Wu et al. [42]. According to this new data, all four swapped segments in our chimeras included some residues corresponding to the S4-S5 linkers (right of cylinders indicating S4 segments). The S4 segments of mouse Ca_V_1.2 (Uniprot ID: Q01815) are identical to the human Ca_V_1.2 (Uniprot ID: Q13936), therefore in the alignments only the mouse sequence used here is shown. The S4 segments of Ca_V_3.1 (Uniprot ID: O43497) and Ca_V_3.2 (Uniprot ID: O95180) human channels are also shown for appreciation of differences within Ca_V_3 subfamily members. Sequence alignments were performed with ClustalW using DNAsis MAX 3.0 (Hitachi Solutions, San Bruno, CA).

Chimeras were made by replacing S4 segments of human Ca_V_3.3 or α1I (GenBank accession number AF393329) by the corresponding region of mouse Ca_V_1.2 or α1C (GenBank accession number AY728090). The whole sequence of each segment swapped between both Ca_V_ channels is presented in Fig 1. The naming of chimeras was as follows: chimera IS4C contains the backbone of Ca_V_3.3 but the S4 segment of Domain I was replaced by the corresponding segment of Ca_V_1.2; a similar logic applies to the chimeras IIS4C, IIIS4C and IVS4C. On the contrary, chimera IIS4I contains the backbone of Ca_V_1.2 and the S4 segment of Domain II was replaced by the corresponding segment of Ca_V_3.3. For the IIS4C and IVS4C chimeras, we used two intermediate constructions in pUC19: one, with the 4737 bp fragment of Ca_V_3.3 (*HindIII*-5’-Polylinker/*HindIII*-4737), named here pUC19-a1I-HindIII; and two, with the 2881 bp fragment of Ca_V_1.2 (*SphI* 1463/*SphI* 4343), named pUC19-a1C-SphI. Then, by using PCR-based mutagenesis with *Pfu* Ultra DNA polymerase (Agilent Technologies, Santa Clara, CA) we introduced restriction sites at the 5’ and 3’ ends of S4 segments of both channels. For IIS4, *SpeI* and *BspEI*; and for IVS4, *AgeI* and *XhoI*, respectively. Then, the S4 segments of Ca_V_3.3 were substituted with the corresponding segment in Ca_V_1.2 using the mentioned enzymes in pUC19-a1I-HindIII. Because the insertion of such restriction sites also generated non-wanted mutations in the sequence of the S4 segments, these were corrected with a second round of PCR-based mutagenesis. Once this was done, the chimeric fragments of Ca_V_3.3 containing the IIS4 and IVS4 of Ca_V_1.2 (in pUC19-a1I-HindIII) were then reinserted back to Ca_V_3.3 wild-type (WT) using the *AfeI* fragment (1537 bp) for the IIS4C chimera, and the *BsrGI-HindIII* fragment (685 bp) for the IVS4 chimera.

In contrast, IS4 and IIIS4 chimeras, were engineered using the overlapping extension PCR method [43]. For each chimeric fragment, four overlapping primers and two conventional primers were used. The combination of these primers in six different PCR reactions using Herculase II Fusion DNA polymerase (Agilent Technologies), originated the IS4C and IIIS4C chimeric PCR fragments, which were cloned into the pJET1.2/Blunt vector by the means of the CloneJET PCR Cloning kit (Thermo Fisher Scientific, Waltham, MA). Full length chimeric channels were made by subcloning fragments from pJET1.2/Blunt into a Ca_V_3.3-WT channel. The *ClaI*-*AvrII* fragment (2786 bp) was used for chimera IS4C and the *AvrII*-*BsrGI* fragment (1388 bp) for the IIIS4C.

In the construction of IIS4I chimera, the constructs pUC19-a1I-HindIII and pUC19-a1C-SphI were used again, but in this case the IIS4 segment from Ca_V_1.2 was replaced for that of Ca_V_3.3 channel. Previously, we have inserted an *AfeI* restriction site in pUC19-a1C-SphI, and this was incorporated into Ca_V_1.2 channel with the subcloning of the *BsrGI*-*SacII* fragment (2303 bp). The insertion of the *AfeI* site generated a point mutation that changed the valine at position 682 for an alanine (V682A). This residue is located in the IIS5-IIS6 loop of Ca_V_1.2 α1 subunit. Such mutation did not produce any changes in the biophysical properties of this mutant compared with the Ca_V_1.2-WT channel (data not showed). Finally, the chimeric fragment containing the IIS4 of Ca_V_3.3 was subcloned into Ca_V_1.2 channel by using the *BsrGI*-*AfeI* fragment (407 bp).

Double chimeras were obtained by the cut and paste strategy of single chimeras. For the chimera IS4C-IIS4C, the *AfeI* fragment (1536 bp) generated by the digestion of IS4C chimera was replaced in IIS4C chimera. In the construction of chimera IS4C-IIIS4C, ligation of three fragments was required. Fragment 1 was obtained by digesting IIIS4C chimera with *ClaI*-*BsrGI* enzymes, the 9053 bp fragment contained the IIIS4C chimeric fragment; fragment 2 was generated from the same chimera using *BsrGI*-*NsII* enzymes, producing a 1812 bp fragment; and fragment 3 was generated by the digestion of IS4C chimera with *ClaI*-*NsiI* (2360 bp; containing the chimeric fragment of IS4C). Finally, chimera IIS4C-IVS4C was obtained through ligation of a 11735 bp fragment, generated by digesting chimera IIS4C with *BspEII* (which contains the chimeric fragment of IIS4C), and the 1490 bp fragment obtained by digesting with the same enzyme the IVS4C chimera (containing the chimeric fragment of IVS4C).

Some chimeras were GFP-fused using the Ca_V_3.3-GFP-HA plasmid [44]. This construction encodes for a fusion protein containing the GFP attached to the amino-end of Ca_V_3.3 channel. With no exception, all GFP-fused chimeras were constructed by swapping of *SbfI*-*KpnI* fragment (7227 bp) between this construction and the respective chimera.

Site-directed mutagenesis of Ca_V_3.3 channel was performed by using the overlapping extension PCR method [43]. In this case, each mutant fragment was generated with three PCR reactions carried out using *Deep Vent* polymerase as specified by the manufacturer (New England Biolabs; Ipswich, MA). All PCR fragments were cloned into the pJET1.2/Blunt vector (Thermo Fisher Scientific), and full length mutant channels were made by subcloning the *AfeI* fragment (1536 pb) from pJET1.2/Blunt into a Ca_V_3.3-WT channel. The molecular identity of all constructions was verified by automated sequencing (Biomolecular Research Facility, Instituto de Fisiología Celular, UNAM).

### Cell culture and transfections

Human embryonic kidney (HEK-293) cells were grown in Dulbecco’s modified Eagle’s medium (DMEM) supplemented with 10% fetal bovine serum, 100 U/ml penicillin, and 100 μg/ml streptomycin at 37°C in a CO_2_ incubator. All cell culture reagents were purchased from Gibco-Thermo Fisher Scientific. The HEK-293 cells were transiently co-transfected with plasmid DNA for green fluorescent protein (GFP) using JetPEI transfection reagent (PolyPlus Transfection, Illkirch, France). Transfections of Ca_V_1.2 and its chimeras included β2a and α2δ1 subunits. All transfections were done with 3 μg of total cDNA, and after 24–72 h GFP-positive cells were selected for electrophysiological experiments.

### Patch clamp experiments

Whole-cell Ca^2+^ currents were recorded at room temperature (21–23°C) according to the patch-clamp technique [45] by using an Axopatch 200B amplifier, a Digidata 1320A/D converter, and the pCLAMP 10.4 software (Molecular Devices, Sunnyvale, CA). Currents were usually digitized and filtered at 5 kHz, except for tail currents which were sampled at 100 kHz and filtered at 10 kHz. Whole-cell series resistance and cell capacitance was estimated from optimal cancellation of the capacitive transients with the built-in circuitry of the amplifier, and when required, compensated electrically by 60–70%. Unless otherwise stated, the holding potential (HP) was -100 mV. In some cases, currents were recorded on two channels, one with on-line leak subtraction using the P/-5 method, and the other to evaluate cell stability and holding current. Only leak subtracted data are shown. Cells were bathed in a solution containing (in mM): 5 CaCl_2_; 160 tetraethylammonium (TEA) chloride; and 10 HEPES (pH 7.4; 310–315 mOsm). The internal (pipette) solution contained (in mM): 135 CsCl; 10 EGTA; 4 Mg-ATP; 0.3 Tris-GTP; and 10 HEPES (pH 7.3; 295–300 mOsm).

In order to obtain peak current values and time constant from exponential fits, current recordings were analyzed with Clampfit 10.4; whereas average data (mean ± standard error) was obtained with Excel; finally graphs were plotted with Prism 6.0 software. The voltage dependence of current activation was estimated using a modified Boltzmann function to fit normalized *I-V* data: *I* = *I*_max_ (*V*m−*V*_rev_) / (1 + exp ((*V*1/2 –*V*_m_)*/k*)), where *I* is current, *V*_m_ is the test potential, *V*_rev_ is the apparent reversal potential, *V*_1/2_ is the mid-point of activation, and *k* is the slope factor. Steady-state inactivation relationships (or availability curves) were obtained by fitting averaged data to a standard Boltzmann function: *I* = *I*_max_ */* (1 + exp ((*V*_m_− *V*_1/2_)*/k*)), where *I*_max_ is the maximal current recorded at -30 mV, *V*_1/2_ is the midpoint of steady-state inactivation, and *k* is the slope. All quantitative results are given as the mean ± SEM. Differences in means were tested either with ANOVA followed by Dunnett’s multiple comparison against WT or unpaired two-tailed Student´s *t*-test, and were accepted as significant if *P* < 0.01.

### Confocal microscopy

For subcellular localization of GFP-tagged Ca_V_3.3 channels, HEK-293 cells were transfected with the corresponding α1-subunit, as described above, and plated on FluoroDish 35-mm dishes (World Precision Instruments, Sarasota, FL) for 8 h under standard incubation conditions. Two hours before images were taken, the medium was replaced with 500 μl of fresh DMEM. Plasma membrane was stained with FM4-64 (Invitrogen-Thermo Fisher Scientific) by adding 1 μM directly to the culture dish 5 min before starting image acquisition. All confocal experiments were performed using an Olympus FluoView FV1000 confocal microscope, using a 60X (1.45 NA) oil immersion objective. For the detection of GFP-tagged calcium channels signal an Argon laser was used (excitation, 488 nm; emission 530 nm), and a Helium-Neon laser (excitation, 543 nm; emission 640 nm) for the FM4-64 signal. Confocal images were processed and analyzed with ImageJ software (National Institutes of Health).

### Western blotting

Total protein from transiently transfected HEK-293 cells was extracted 48 h post-transfection using RIPA buffer (25 mM Tris-HCl, pH 7.4; 150 mM NaCl; 1% IGEPAL; 1% Sodium deoxycholate, and 1% SDS) supplemented with complete EDTA-free protease inhibitors (Roche, Switzerland), and quantified by Bradford assay. Equal amounts of protein (30 μg) were subjected to SDS-PAGE, transferred onto a polyvinylidene difluoride membrane (Millipore, Billerica, MA) and probed overnight with a specific polyclonal anti-GFP antibody at a 1:3000 dilution (Santa Cruz Biotechnology, Dallas, TX). Blots were subsequently probed with anti-rabbit secondary antibody conjugated with horseradish peroxidase (1:10000; Santa Cruz Biotechnology), and visualized using the SuperSignal West Pico chemiluminescent substrate (Thermo Fisher Scientific). To confirm equal protein loading, blots were striped and probed with a homemade anti-β-actin antibody diluted 1:1000. Signal intensity of immunoblots was calculated with ImageJ software.

## Results

### Effects of S4 segments on the low-voltage activation of Ca_V_3.3 channels

**Fig 2:**
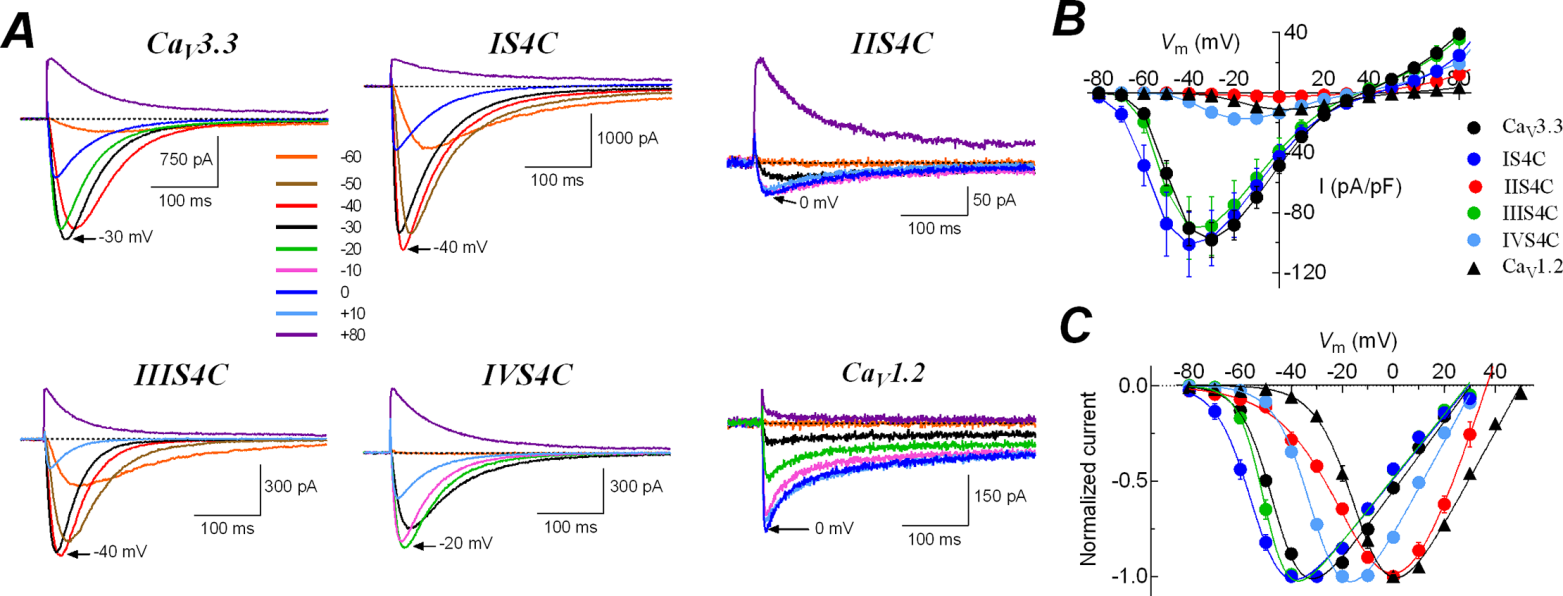
Contribution of S4 segments to the low-voltage activation of Ca_V_3.3 channels. ***A***, representative calcium currents of Ca_V_3.3, IS4C, IIS4C, IIIS4C, IVS4C, and Ca_V_1.2 channels at the indicated voltages. Holding potential was -100 mV, except for recordings of IS4C, where it was -120 mV. Charge carrier was always 5 mM Ca^2+^. The voltage of maximal inward current for each channel is indicated by arrows. Note the very tiny inward currents generated by IIS4C chimera, compared with those of Ca_V_3.3 channel. ***B***, current-voltage (*I-V*) relationships. Peak current amplitudes were normalized to the *C*_m_ value of each cell, averaged, and plotted as a function of test potential. Note that current densities were very small for IIS4C, IVS4C, and Ca_V_1.2 channels. ***C***, normalized *I-V* curves. For clarity purposes only inward currents are plotted. Solid lines are the best fits to the data with a modified Boltzmann function (see Material and Methods). Same cells as shown in ***B***. Data in graphs represent mean ± SEM. Parameter values, number of cells and statistical significance are shown in Table 1.

We have investigated the role of the four S4 segments of the α1 subunit in the low-voltage activation behavior of Ca_V_3.3 (α1I) channels. As in previous studies [46,47], we used HEK-293 cells, a mammalian cell line, to express the constructed chimeras and Ca^2+^ as charge carrier to perform whole-cell patch-clamp experiments. In order to investigate the role of each of the voltage sensors in the activation of Ca_V_3.3 channels we replaced the S4 segments of this channel with those of Ca_V_1.2 (Fig 1). In all our experiments, Ca_V_3.3 α1 subunit was transfected alone, whereas Ca_V_1.2 channels were co-transfected with β2a/α2δ1 auxiliary subunits. As previously reported [34,48], Ca^2+^ currents of Ca_V_3.3-WT channels are transient and activated at low voltages, while calcium currents of Ca_V_1.2 channels are more sustained and activated at more depolarized potentials (Fig 2A). The maximal inward current for Ca_V_3.3 channels was observed at -30 mV and at 0 mV for Ca_V_1.2 channels, and currents were significantly larger for the LVA channel (Fig 2B). The normalized curves (Fig 2C) show the low-voltage activation characteristic of Ca_V_3.3 channels (black circles), compared with the ~30 mV right-shifted curve of Ca_V_1.2 (black triangles). The potential for the mid-point of activation (*V*_1/2_) and the slope of the relationship (*k*) was -45.4 ± 0.7 mV, and 6.0 ± 0.1 mV for Ca_V_3.3-WT channels, and -11.9 ± 1.3 mV, and 7.4 ± 0.3 mV for Ca_V_1.2 channels (Table 1). As expected, there was a difference in the *V*_1/2_ of around 35 mV, which defines the difference in the voltage-dependence of activation between LVA and HVA channels.

**Table 1:** Activation parameters of Ca_V_3.3, Ca_V_1.2 and chimeric channels.

Channel	pA/pF	*V*_1/2_ (mV)	*k* (mV)	*V*_rev_ (mV)	*n*
Ca_V_3.3	-98.3 ± 11.5	-45.4 ± 0.7	6.0 ± 0.1	30.1 ± 0.5	39
IS4C	-97.1 ± 18.4	-53.8 ± 1.5^*b*^	6.5 ± 0.3	29.7 ± 0.8	11
IIS4C	-2.7 ± 0.4^*a*^	-7.8 ± 2.2^*a*^	12.4 ± 0.8^*a*^	38.5 ± 1.5^*a*^	21
IIIS4C	-90.2 ± 20.9	-49.8 ± 0.9^*b*^	4.8 ± 0.2^*b*^	29.0 ± 0.6	10
IVS4C	-17.6 ± 3.7^*a*^	-29.6 ± 0.8^*a*^	6.9 ± 0.1^*b*^	32.1 ± 0.7	14
IS4C-IIS4C	-2.6 ± 0.6^*a*^	-5.6 ± 5.9^*a*^	13.2 ± 0.5^*a*^	38.2 ± 1.4^*a*^	9
IS4C-IIIS4C	-36.6 ± 6.1^*a*^	-51.0 ± 1.2^*b*^	5.6 ± 0.3	26.2 ± 0.4^*b*^	9
IIS4C-IVS4C	-0.4 ± 0.1^*a*^	-3.8 ± 4.9^*a*^	15.4 ± 1.9^*a*^	38.19± 6.3^*a*^	5
IIS4I	-10.4 ± 2.3	-27.1 ± 2.1^*c*^	7.0 ± 0.3	42.6 ± 2.8^*c*^	16
Ca_V_1.2	-11.3 ± 2.7	-11.9 ± 1.3	7.4 ± 0.3	50.3 ± 1.7	16

Values are given as mean ± SEM. Current density (pA/pF) from *I-V*’s peak. *V*_1/2_, *k* and *V*_rev_ values were obtained from *I-V* data fits from each cell with a modified Boltzmann equation (see Experimental Procedures), and then averaged. The number of investigated cells is shown in the *n* column. Statistical significance is indicated with ^*a*^ or ^*b*^ when using analysis of variance followed by Dunnett’s multiple comparison against WT (*P* < 0.01) or Student’s *t* test (*P* < 0.01), respectively, for Ca_V_3.3 as a control, and ^*c*^ when using Student’s *t* test (*P* < 0.01) for Ca_V_1.2 as control.

Then, we characterized the voltage-dependence of activation of each of the four Ca_V_3.3 chimeras to evaluate their contribution to the LVA behavior of Ca_V_3.3 channels. As shown in Fig 2A, the four chimeras of the Ca_V_3.3 channel, IS4C, IIS4C, IIIS4C, and IVS4C, were functionally expressed in HEK-293 cells and generated robust inward currents, except for IIS4C, which displayed currents than were even smaller than the Ca_V_1.2 channel. In order to eliminate cell size as a variable, we normalized row current values to the membrane capacitance (*C*_m_) for each cell, and the ratio is reported as current density for each cell. Current density was 98.3 ± 11.5 pA/pF for Ca_V_3.3-WT channels, in contrast, this value was almost 9-times smaller for the Ca_V_1.2 channel, and still smaller (around 36 times) for the IIS4C chimera (Table 1). Although, is not unusual that calcium channels mutants or chimeras result in smaller current densities compared with the wild-type channel, due to the characteristics of this chimera we further study the likely causes of this small current (see below).

Regardless the differences observed in current density for each chimera, the striking results were obtained with chimera IIS4C in which the voltage sensor of domain II (IIS4 segment and part of the S4-S5 linker; Fig 1) of Ca_V_3.3 channel was replaced with the corresponding segment of Ca_V_1.2. It displayed the most right-shifted relationship (more than 35 mV), almost overlapping the corresponding *I-V* curve of Ca_V_1.2 channels (Fig 2C). Fitting of IIS4C experimental data with a modified Boltzmann function gives a *V*_1/2_ value of -7.8 ± 2.2 mV, practically the same of Ca_V_1.2 channels (Table 1). We use this method to calculate activation parameters in order to allow direct comparison with previous studies, however, strictly speaking is invalid to use a modified Boltzmann function since open Ca_V_ channels do not obey Ohm´s linear law. Therefore, the parameters of Table 1 should be considered as empirical measurements that may not fully describe the true voltage dependence of the activation process. Nevertheless, the meaningful of the shift in *V*_1/2_ observed for the IIS4C chimera in comparison with the Ca_V_3.3-WT channel (Table 1), was also confirmed when using a tail currents protocol, where the tail current amplitude was plotted versus the voltage of 20-ms depolarizing pulses (S1 Fig). This activation analysis also showed a much smaller change in the slope’s relationship (*k* parameter) for the activation of IIS4C chimera when compared with the WT channel (less than 2 mV; S1 Fig), whereas the modified Boltzmann function method yield a difference of 6 mV (Table 1). Therefore, the results regarding the *k* parameter of activation curves shown in Table 1 should be taken cautiously, as they may not accurately reflect the changes in the slope´s relationships. Finally, the chimera IIS4C was followed by IVS4C chimera in terms of displaying HVA-like *I-V* curves, with about a 15 mV shift to more positive voltages (Fig 2C); and the swapping of Domains I and III voltage sensors (chimera IS4 and IIIS4), induced smaller, but significant, shifts in the *I-V* curve to more negative potentials (Table 1).

The results of swapping the voltage sensors of each domain of Ca_V_3.3 channels with those of Ca_V_1.2 channels show a crucial role of Domain II voltage sensor and, in to a lesser degree the Domain IV voltage sensor, for the low-voltage activation behavior of these channels. We further investigated the influence of those segments by constructing double chimeras; a total of three additional chimeras were engineered and constructed: chimera IS4C-IIS4C, in which IS4 and IIS4 segments of Ca_V_3.3 were swapped for those of Ca_V_1.2 channel; and chimeras IS4C-IIIS4C and IIS4C-IVS4C, following the same rationale. The results for the voltage-dependence of activation of these chimeric channels are summarized also in Table 1. As can be appreciated, the *V*_1/2_ value for the chimera IS4C-IIS4C was practically the same as for the IIS4 chimera alone, meaning that the contribution of IIS4 segment overpassed the effect observed for the IS4C chimera, which alone induced a shift in the *V*_1/2_ value toward more negative potentials compared with the Ca_V_3.3-WT channel (around 8 mV; Table 1). Also the parameters *k* and *V*_rev_ were pretty similar to those observed for the IIS4C chimera alone, rather than to the IS4C chimera (Table 1), which indicates that both chimeric channels were less voltage dependent than the Ca_V_3.3 channel. On the other hand, when the IS4C construction was combined with chimera IIIS4C, i.e., IS4C-IIIS4C double chimera; the parameters of voltage-dependence of activation were closer to the IIIS4C chimera (Table 1). The third double chimera, IIS4C-IVS4C, behaved as predicted by the results of individual IIS4C and IVS4C chimeras, that is, the currents were activated at very positive potentials compared with the Ca_V_3.3-WT channels, meaning that those two voltage sensors (from domains II and IV), have the major contribution to the low-voltage activation of Ca_V_3.3 channels. The double chimera displayed a *V*_1/2_ value even more positive than the Ca_V_1.2 channel itself, although the difference was not significant at *P*<0.01. On the contrary, the steepness of the *I-V* curve decreases drastically with a slope value of 15.4 ± 1.9 mV, even more than IIS4C alone (12.4 ± 0.8 mV), and almost 3-times larger than that of Ca_V_3.3 wild-type channel (Table 1). In addition, the current density was so low, yet detectable (less than 1 pA/pF) that only the *I-V* voltage protocol produced reliable data to characterize this double chimera. In summary, the results presented in this section show that voltage sensors of domain II and IV have a substantial contribution to the low-voltage activation behavior of Ca_V_3.3 channels, although individually the IIS4C chimera induced the most significant changes.

### Effects on current activation and inactivation kinetics

**Fig 3:**
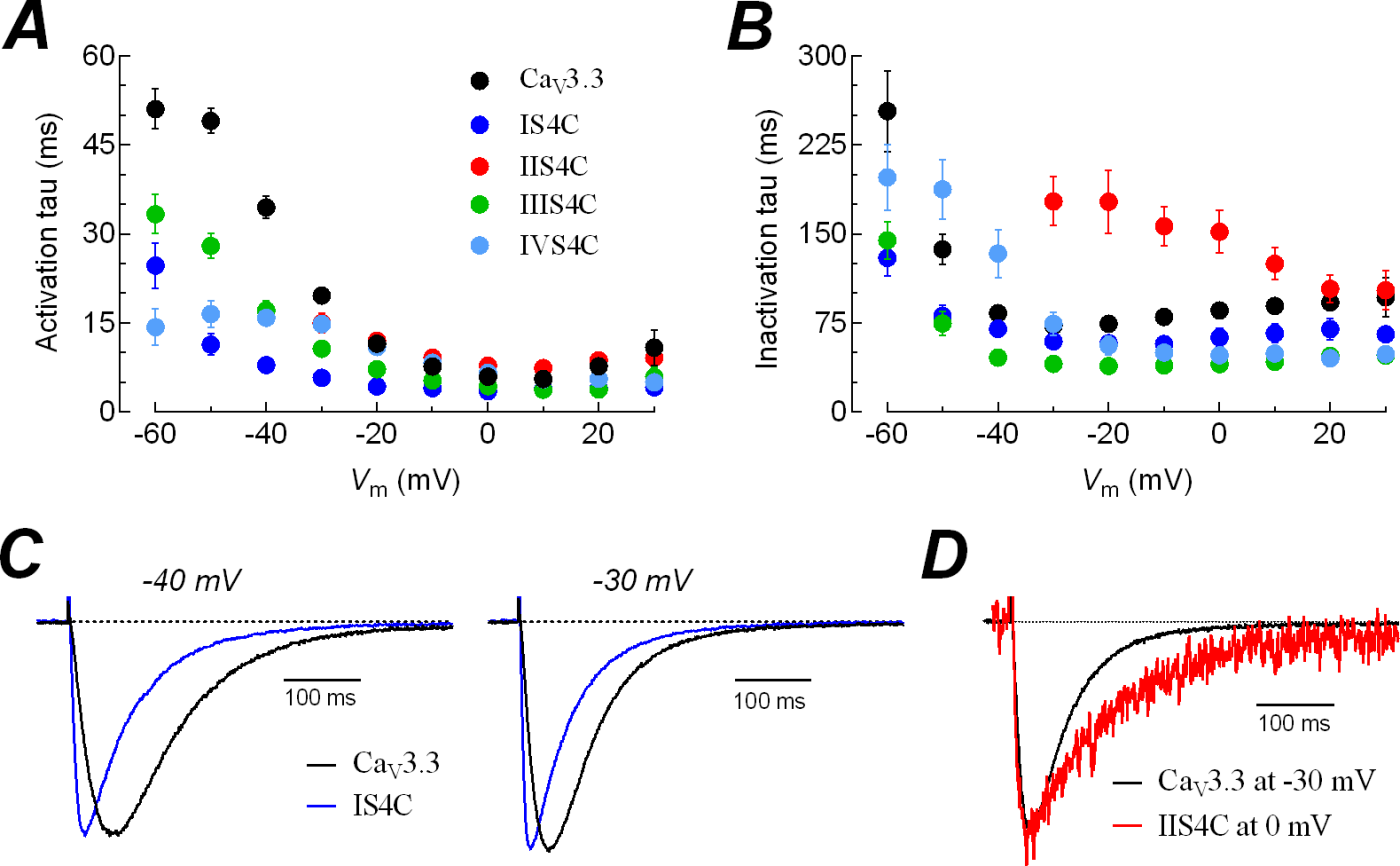
Time course of activation and inactivation of the current from Ca_V_3.3 channels and the chimeras. Time constants (tau) of activation (***A***) and inactivation (***B***) for Ca_V_3.3 and the indicated chimeric channels. Currents like those illustrated in Fig 2*A*, were fitted with two exponentials, one for the activation and the other for the inactivation of the current; and the respective constants were plotted as a function of membrane potential. Due to the small amplitude and the 30 mV shift in the *I-V* curve of IIS4C chimera (Fig 2*A* and 2*B*), data of fitted currents is only shown for the range of -30 to +30 mV. Data points in graphs represent mean ± SEM; same cells as in Fig 2*B*. ***C***, representative current traces showing the much faster activation kinetics for the IS4C chimeric channel than Ca_V_3.3 channels at -40 and -30 mV. Peak currents of Ca_V_3.3 were normalized to those of IS4C channel (x1.49 and x1.29, respectively). ***D***, inactivation kinetics of IIS4C channel are slower than the Ca_V_3.3-WT channel. Calcium currents recorded at -30 mV for Ca_V_3.3 and at 0 mV for IIS4C channels. Chimeric IIS4C peak current was scaled up to that of Ca_V_3.3 channel by a 21.7 factor.

We next sought to examine the effects of swapping the voltage sensors on current kinetics. The effects were observed mainly in the time course of current activation of IS4C, IIIS4C and IVS4C chimeras, being drastically accelerated in comparison with the Ca_V_3.3-WT currents; and significantly slowed down inactivation kinetics of IIS4C chimera (Fig 3A and 3B). For all chimeras, smaller activation tau values were observed in the range from -60 to -20 mV. In this case, the IS4C chimera showed the more drastic effect; for example, the activation tau at -30 mV was 19.6 ± 1.1 ms and 5.8 ± 0.7 ms, for the wild-type and the chimeric channel, respectively. In other words, current expressed by the IS4C chimera activates with a time course more than 3-fold faster than the wild-type channel. In fact, this difference can be observed in the normalized currents recorded at -40 and -30 mV of these two channels illustrated in Fig 3C. On the contrary, the major difference of the IIS4C chimera with the Ca_V_3.3 current kinetics was observed in the tau constant of inactivation, which was significantly slowed down. At -30 mV, inactivation of the chimeric channel was 2.5-times slower than the Ca_V_3.3 channel (Fig 3B); and considering the 35 mV shift in the IV of both channels (Fig 2C), still the inactivation of the chimeric channel was twice slower than the Ca_V_3.3 channel (72.5 ± 3.5 ms at -30 mV for Ca_V_3.3 and 151.9 ± 18.0 ms at 0 mV for IIS4C chimera; *P<0.01; Fig 3D). An opposite effect was observed for chimeras IS4C and IIIS4C, which showed faster inactivation kinetics (mainly between -20 to +20 mV) than the Ca_V_3.3 channel (Fig 3B). The most drastic effect was observed with the IIIS4C chimera that exhibited a tau value of 40.4 ± 2.6 ms at 0 mV, compared with 85.7 ± 4.3 ms for the WT-channel (*P<0.01). The IS4C current inactivation was faster too, although to a lesser degree (Fig 3B). Thus, the IIS4 voltage sensor is also relevant for the inactivation of Ca_V_3.3 channel as the chimera significantly slowed down the transition rate of open to inactivated channels, whereas the IIIS4C and IVS4C chimeras accelerated the same transition. Also, activation is severely accelerated by the IS4C, indicating that this voltage sensor is important for the transition from the closed to the open state, but with less influence in the inactivation from the open state of Ca_V_3.3 channels. Similar results were obtained for the activation and inactivation kinetics of chimeras between voltage sensors of Ca_V_3.1 and Ca_V_1.2 channels [29].

A couple of previous reports with a similar topic of the present research did not explore the effects of swapping voltage sensors between LVA and HVA channels beyond the voltage-dependence of activation and the associated current kinetics [29,30]. On the contrary, in this study we investigated whether additional distinctive properties of LVA channels were modified in the chimeric Ca_V_3.3 channels, in particular the steady-state inactivation, the recovery from inactivation and the closing time of the channels.

### The role of S4 segments in inactivation and recovery at negative potentials of Ca_V_3.3 channels

**Fig 4:**
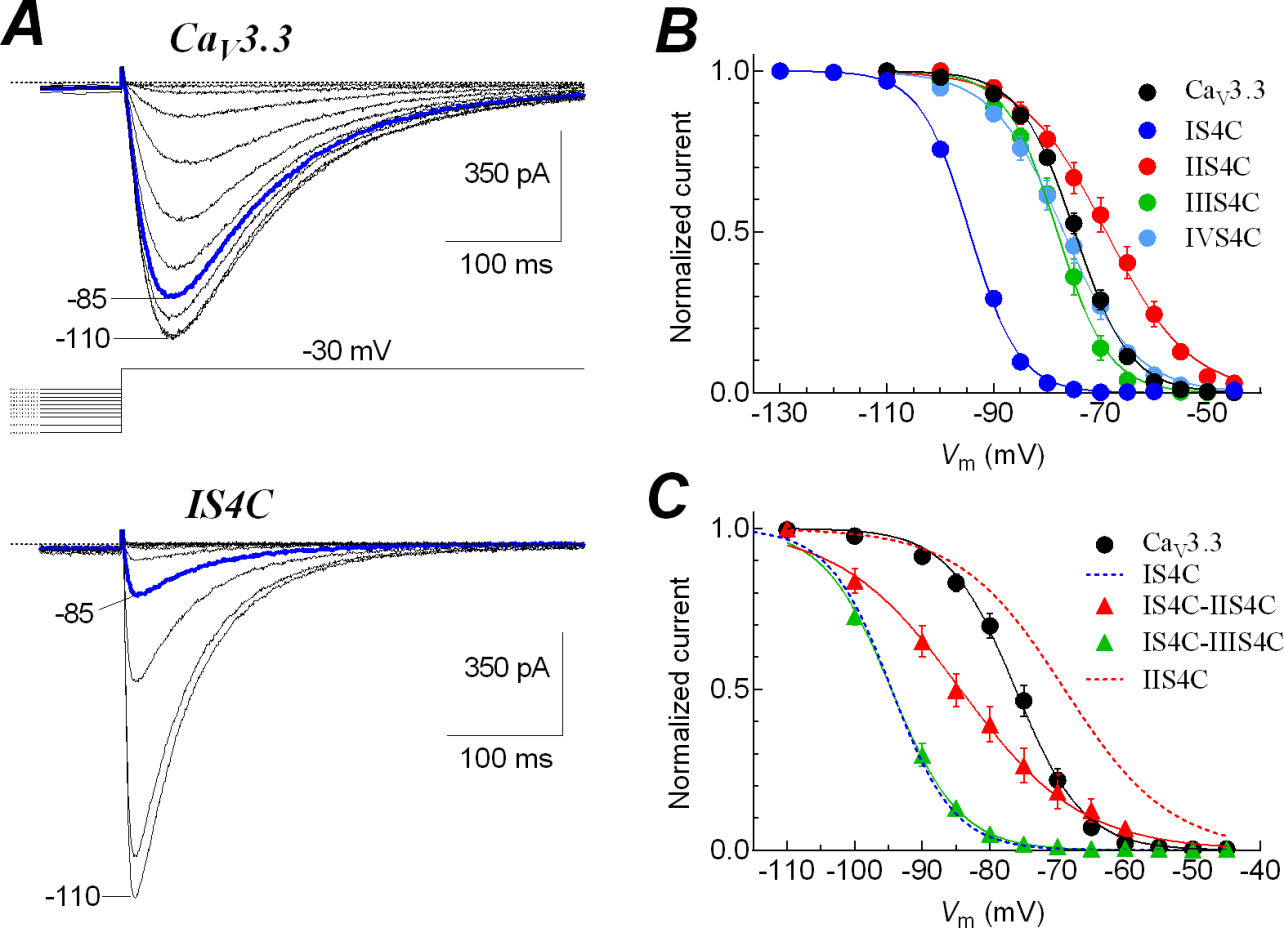
Contribution of S4 segments to the steady-state inactivation of low-voltage activated Ca_V_3.3 channels. ***A***, families of currents recorded at -30 mV from Ca_V_3.3 (upper traces) or IS4C chimeric (bottom traces) channels. Current recordings were obtained after 15-s prepulses to potentials between -110 and -45 mV, as shown by the voltage protocol. For a better appreciation only the last 70 ms of the prepulses are shown. Blue traces show the difference in voltage channels availability after the prepulse to -85 mV in both channels. ***B***, steady-state inactivation curves for WT and chimeric Ca_V_3.3 channels. Currents at -30 mV like those shown in ***A*** (or at 0 mV for IIS4C) were normalized to the value at -110 (-130 for IS4C) mV, averaged and plotted as a function of the prepulse potential for each channel. Smooth lines are fits to Boltzmann functions. ***C***, steady-state inactivation curves for Ca_V_3.3 and its double-chimeras IS4C-IIS4C and IS4C-IIIS4C. Currents at -30 mV like those shown in ***A*** (or at 0 mV for IS4C-IIS4C) were normalized to the value at -110 mV, averaged and plotted as a function of the prepulse potential for each channel. Smooth lines are fits to Boltzmann functions. Data in graphs represent mean ± SEM. Parameter values, number of cells and statistical significance are shown in Table 2.

The steady-state inactivation at more hyperpolarized potentials is an additional property of LVA channels compared with HVA channels, whose inactivation take place at more depolarized potentials [49,50]. As expected, the Ca_V_3.3-WT channels showed a typical decrease in channel availability at very negative potentials (Fig 4A, upper traces), and this behavior was not significantly affected by the replacement of IIIS4 and IVS4 voltage sensors with those of Ca_V_1.2 channel. In contrast, the substitution of the IIS4 voltage sensor (chimera IIS4C) lead to a significant shift in the steady-state inactivation curve toward more depolarized potentials (around 6 mV; *P<0.01, Table 2; Fig 4B). The change in the mid-point inactivation potential (*V*_1/2_) was also accompanied by a significant change in the steepness of the inactivation curve, parameter *k*, from 4.8 ± 0.1 mV in Ca_V_3.3 to 8.4 ± 0.6 mV in the IIS4C chimeric channels (*P<0.01, Table 2), inducing again an HVA-like behavior of the channel, and a decrease in the voltage-dependence of inactivation. Somewhat unexpected, the chimeric IS4C channels inactivated at potentials much more negative than the Ca_V_3.3 channels (Fig 4A, bottom traces). While the channel availability after a 15-s prepulse to -85 mV was around 85% for wild-type, in the chimeric IS4C channel this value was diminished to less than 10% (compare blue traces of Fig 4A). As a consequence, the steady-state inactivation curve of IS4C chimera was shifted around 20 mV toward more negative potentials in comparison with the Ca_V_3.3 channel (Fig 4B). This drastic difference in the *V*_1/2_ values of inactivation occurred without significant changes in the slope of the relationships (parameter *k*; Table 2), suggesting that the rate of inactivation from closed states was the same for both channels, the difference being that the IS4C chimera changed the voltage range where such inactivation occurs. Therefore, the fundamental contribution of IS4 voltage sensor for preventing inactivation of Ca_V_3.3 channels at more negative potentials was further evidenced with the IS4C-IIS4C double chimera, whose steady-state inactivation curve was significantly shifted to more negative potentials in comparison with that one of individual IIS4C chimera, and of course, with the one of Ca_V_3.3 wild-type curve (Fig 4C). Also, the steady-state inactivation curve of IS4C-IIIS4C double chimera was fitted with *V*_1/2_ and *k* parameters very similar to those obtained from the IS4C individual chimera (Table 2). In other words, the contribution of IS4 voltage sensor to the inactivation of Ca_V_3.3 channels is so critical that it overrode the high-voltage activation behavior induced by the IIS4C chimera.

**Table 2:** Steady-state inactivation parameters of Ca_V_3.3, Ca_V_1.2 and chimeric channels.

Channel	*V*_1/2_ (mV)	*k* (mV)	*n*
Ca_V_3.3	-74.6 ± 0.6	4.8 ± 0.1	32
IS4C	-93.3 ± 1.4^*a*^	4.0 ± 0.3	7
IIS4C	-68.4 ± 1.7^*b*^	8.4 ± 0.6^*a*^	12
IIIS4C	-77.9 ± 1.0	4.6 ± 0.1	8
IVS4C	-76.5 ± 1.3	6.2 ± 0.4^*b*^	14
IS4C-IIS4C	-83.3 ± 2.2^*a*^	9.0 ± 0.8^*a*^	7
IS4C-IIIS4C	-94.6 ± 0.7^*a*^	4.8 ± 0.1	8
IIS4I	-79.3 ± 1.5^*c*^	9.3 ± 0.8	8
Ca_V_1.2	-36.0 ± 5.4	12.2 ± 1.5	8

Values are given as mean ± SEM, and were obtained from Boltzmann function fits to steady-state inactivation data for each cell and then averaged. The number of investigated cells is shown in the *n* column. Statistical significance is indicated with ^*a*^ or ^*b*^ when using analysis of variance followed by Dunnett’s multiple comparison against WT (*P* < 0.01) or Student’s *t* test (*P* < 0.01), respectively, for Ca_V_3.3 as a control, and ^*c*^ when using Student’s *t* test (*P* < 0.01) for Ca_V_1.2 as control.

**Fig 5:**
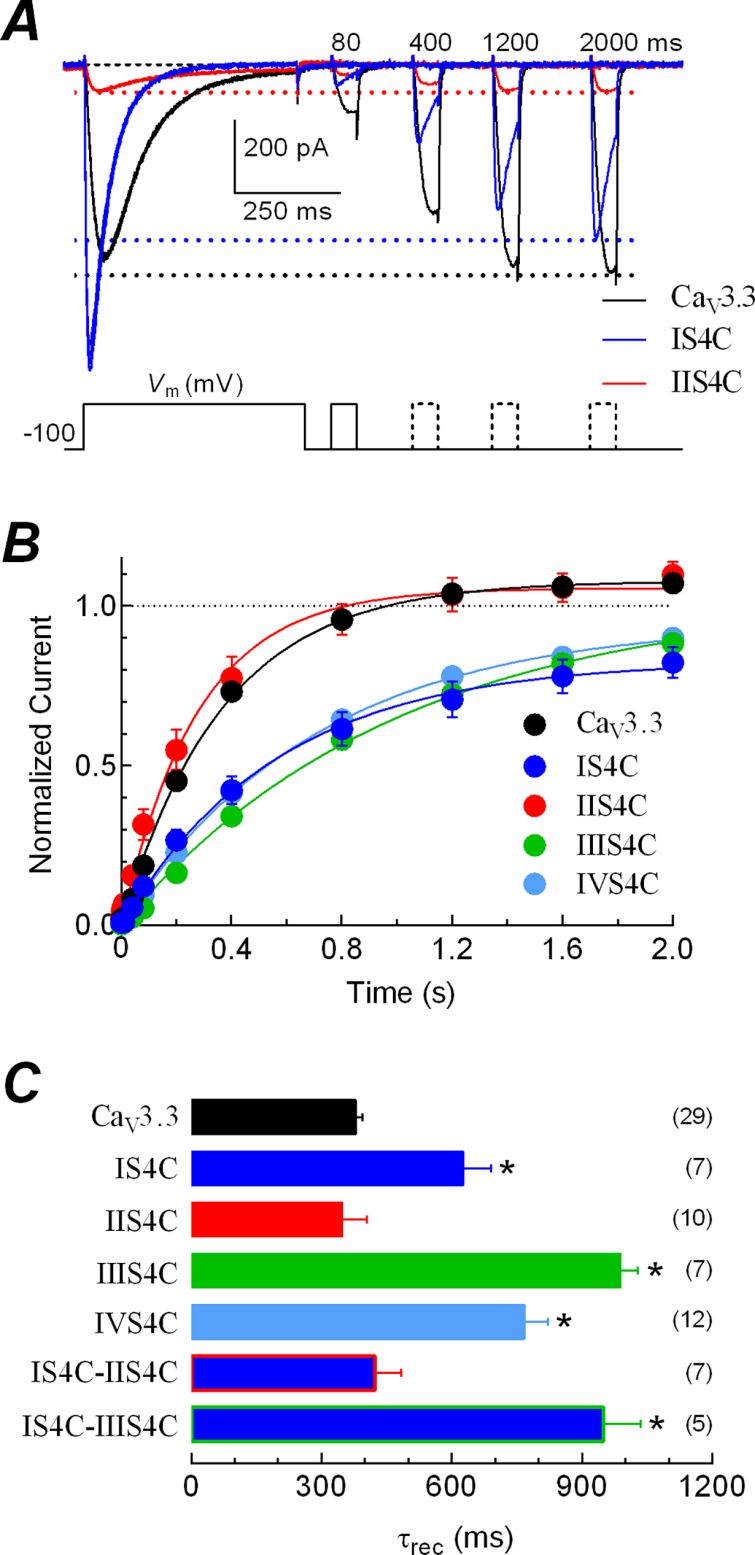
Consequences on the recovery from inactivation of Ca_V_3 channels due to the substitution of S4 segments. ***A***, representative recordings of Ca^2+^ currents illustrating the recovery from inactivation of Ca_V_3.3, IS4C and IIS4C channels. As shown by the two-pulse protocol at the bottom, calcium currents were inactivated by a 500 ms pulse to -30 mV (-10 for IS4C), then the membrane potential was stepped to -100 mV for periods ranging from 1 to 2000 ms before applying a 60 ms activating voltage step to -30 mV (-10 for IS4C). Shown are traces corresponding to 0.08, 0.4, 1.2 and 2 s between the turn off of the inactivating pulse and the turn on of the test pulse. Tail currents generated by the membrane repolarization to -100 mV were cut out to emphasize the amplitude of the currents. Dashed lines indicate the maximum current amplitude recovered after 2 s at -100 mV for each channel. ***B***, time course of recovery from inactivation at -100 mV for the indicated Ca_V_3.3 channels. Data are the peak current (mean ± SEM) during the 60-ms pulse, normalized to the peak current recorded during the 500-ms pulse. Smooth curves are fits to the data using a one phase exponential association equation. ***C***, time constants of recovery from inactivation (τ_rec_). Columns are means, and bars the standard error. Number of investigated cells are given in parenthesis. *Statistical significance with one-way analysis of variance followed by Dunnett’s multiple comparison test against Ca_V_3.3. Note that the fraction of IS4C chimeric channels recovered after 2 s was incomplete and slower than Ca_V_3.3 channels. On the contrary, IIS4C channels recovered faster and at the same level of wild-type channels.

Both parameters, the speed of channel inactivation from the open state (Fig 3B), and the voltage-dependence of inactivation from closed state (Fig 4B and 4C), were substantially modified by the chimeras. Therefore, significant changes in the recovery from inactivation caused by the substitutions of the S4 voltage sensors could be predicted. Representative currents illustrating the recovery of Ca_V_3.3, IS4C and IIS4C channels at -100 mV are shown in Fig 5A. As shown previously [34,51], Ca_V_3.3 current recovered to a value slightly greater than control (after 2 s at -100 mV, normalized current was 1.07 ± 0.01; *P* < 0.01), because of the facilitation induced by the inactivating prepulse. On the contrary, the fraction of current recovered after the same period of time was incomplete for the IS4C channels (indicated by the blue dashed line) than for the Ca_V_3.3 and IIS4C channels (black and red dashed lines). Relationships of normalized current amplitudes versus time for the single chimeras and the Ca_V_3.3 channels are shown in Fig 5B. In addition to the incompleteness of current recovery, chimeras IS4C, IIIS4C and IVS4C exhibited a slower time course of recovery from inactivation than the Ca_V_3.3 and the IIS4C chimeric channels, in fact, the latter channels showed a slightly faster (not significant) tau of recovery (τ_rec_; Fig 5C). The effect of partial recovery was more pronounced in the IS4C chimera as the fractional recovery reach a value around 84 ± 4% (*n* = 7) after 2 seconds, whereas the IIIS4C and IVS4C chimeras still show a tendency to increase the fractional recovery at the same time (Fig 5B). The apparent incompleteness of recovery from inactivation for these chimeras is not due to an irreversible rundown of the current during the lasting of the voltage protocol, but instead to a real slower component of recovery induced by the swapping of the respective voltage sensors. This is based on the fact that the amplitude of the peak current evoked by the first (at 0 s) and last prepulse (at 2 s) of the applied voltage protocol (illustrated in Fig 4A) were practically the same: Ipeak pp 2s/Ipeak pp 0s was 0.96 ± 0.08 for IS4C; 0.97 ± 0.03 for IIIS4C; and 1.09 ± 0.05 for IVS4C. In a few cells we actually applied longer protocols finding that after 6 s, these chimeras were able to fully recover from inactivation. Tau of recovery was significantly slower for IS4C, IIIS4C and IVS4C, and also for the double chimera IS4C-IIIS4C; but not for the IS4C-IIS4C chimera (Fig 5C), evidencing again the major influence of the IIS4 voltage sensor over the IS4 on the speed for the recovery from inactivation of the channels. However, for the completeness of recovery, the IS4C chimera was preponderant over the IIS4C channels, as the double chimera IS4C-IIS4C reached a plateau recovery of 90 ± 3% (*n* = 7); which was not statistically different from the IS4C single chimera (84 ± 4%; *n* = 7; *P*<0.001), but the difference was significant compared with the IIS4C single chimera (108 ± 4%, *n* = 10, *P*<0.001). The results of the recovery from inactivation are in agreement with the alterations observed in the inactivation from the open state and the shift of the steady-state inactivation curve for such chimeras, suggesting a differential role of the S4 voltage sensors in such biophysical properties of the Ca_V_3.3 channels.

### Effects of S4 segments on the slow deactivation behavior of Ca_V_3.3 channels

**Fig 6:**
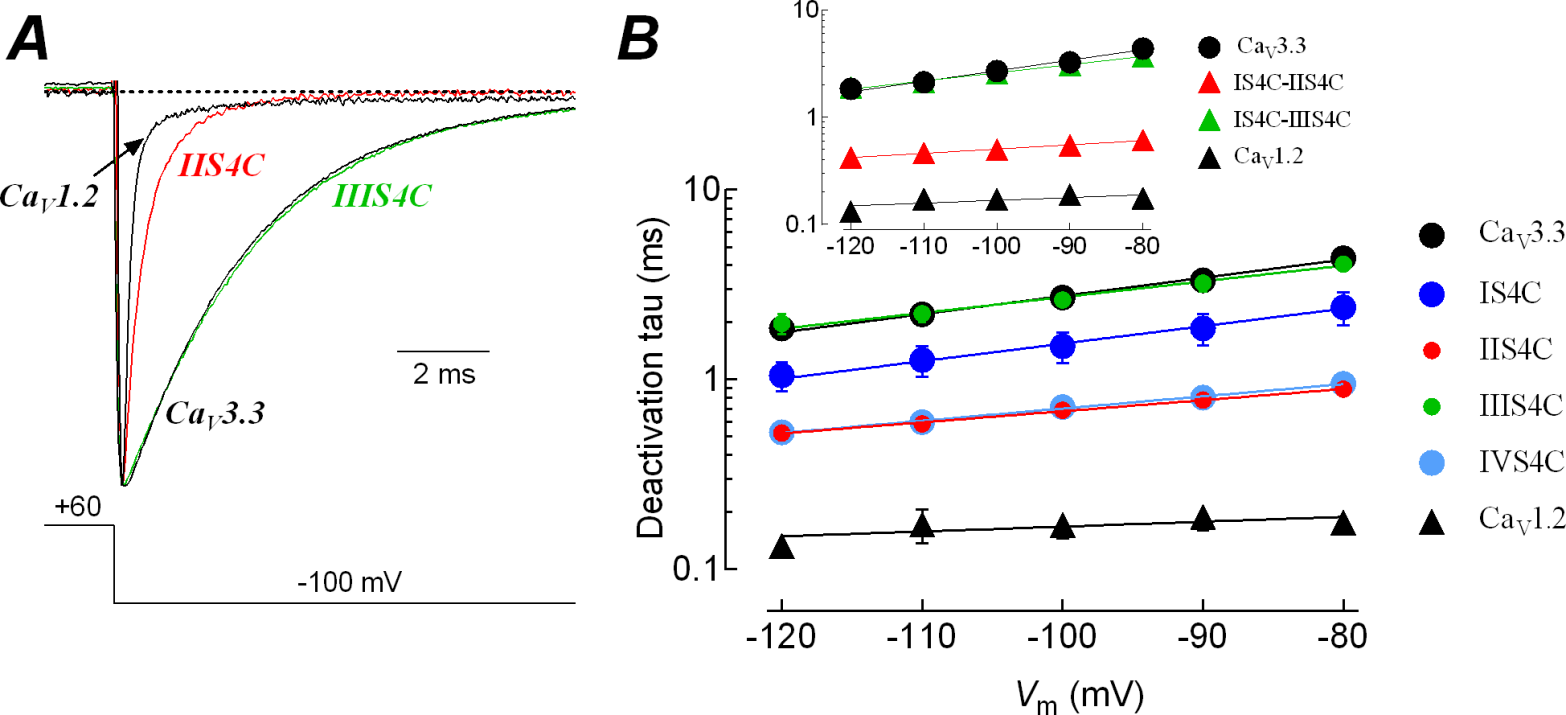
Effect of S4 segments in the slow-deactivating behavior of Ca_V_3.3 channels. ***A***, representative tail currents for Ca_V_3.3, IIS4C, IIIS4C and Ca_V_1.2 channels. Ten-ms depolarization pulses to +60 mV were used to activate channels, and deactivation was recorded during subsequent repolarization to -100 mV, as indicated. Tail current amplitudes were scaled to Ca_V_3.3 tail amplitude. For clarity, the voltage protocol and current recordings show only 1.5 out of 10 ms of the depolarization to +60 mV, and 10 out of 30 ms of the repolarization. ***B***, time constants of channel deactivation for the indicated channels as a function of the repolarizing potential. Time constants were estimated by fitting tail currents to a single exponential function. Data in graphs represent mean ± SEM. For clarity purposes, size of IIS4C and IIIS4C data points have been reduced to show the similarity with those of IVS4C and WT channels, respectively. For example, time constant values at -90 mV were: 3.33 ± 0.15 ms for Ca_V_3.3 (*n* = 25); 1.86 ± 0.35 ms for IS4C (*n* = 7); 0.81 ± 0.03 ms for IIS4C (*n* = 16); 3.22 ± 0.32 ms for IIIS4C (*n* = 6); 0.80 ± 0.06 ms for IVS4C (*n* = 12); and 0.19 ± 0.03 ms for Ca_V_1.2-WT (*n* = 7). Smooth lines are single exponential fits to the averaged tau values. *Inset*: Time constants of channel deactivation for the double chimeras and Ca_V_3.3 channels. The IS4 segment is not relevant for the slow closing of Ca_V_3.3 channel, as double chimeras with IIS4C or IIIS4C follows the individual behavior of these two: fast closing with first and slow closing with the second one. The number of studied cells was 7 for each channel.

Another distinctive characteristic between HVA and LVA channels is the 10- to 20-fold slower deactivation of the Ca_V_3 channels in response to the repolarization of the membrane potential [52,53]. Such a difference can be observed in the tail currents illustrated in Fig 6A. After a 10-ms activating pulse to +60 mV from a HP of -100 mV, the repolarization to -100 mV generated tail currents that were very fast for the Ca_V_1.2-WT channels (deactivation tau, τ_Deact_ = 0.17 ± 0.03 ms, *n* = 7), but rather slow for the Ca_V_3.3-WT channels (τ_Deact_ = 2.7 ± 0.12 ms, *n* = 25). Again the IIS4C chimera showed a behavior very similar to the Ca_V_1.2 channel, exhibiting tail currents that were 4-times faster (τ_Deact_ = 0.69 ± 0.05 ms, *n* = 9) than the Ca_V_3.3-WT channels, almost overlapping with that of Ca_V_1.2 channel. On the contrary, the tail current of the IIIS4C chimera overlapped almost perfectly with the one of the Ca_V_3.3-WT channel. These differences were observed for a range of voltages between -120 and -80 mV (Fig 6B). The chimeric IVS4C channels also deactivated with a much faster time course (τ_Deact_ = 0.71 ± 0.05 ms, *n* = 12), whereas the closing of IS4C chimera develops with intermediate constant tau values (τ_Deact_ = 1.66 ± 0.18 ms, *n* = 5) (Fig 6B). The double chimera IS4C-IIS4C also imposed the HVA fast-deactivating behavior on the Ca_V_3.3 channel, and the IS4C-IIIS4C closed as slow as the Ca_V_3.3-WT channel (Fig 6B, Inset). The acceleration in tail currents observed in IIS4C and IVS4C channels could not be due to the positive shift of activation observed for these channels (Fig 2C, Table 1), as the voltage-dependence of deactivation time constants does not show a parallel shift along the voltage axis (Fig 6B). In addition, as previously described for Ca_V_3 channels [51,54], the final step for channel opening (*k*_O_) is voltage independent, in contrast with closing of the channel (*k*_-O_) which is strongly voltage dependent. Thus, any modification in the tail currents kinetics (closing of the channel) is not related to the voltage of channel activation (opening), but to the modification of the constant rate of closing, *k*_-O_; therefore, the shift in the *V*_1/2_ of activation of IIS4C chimeric channels does not explain the acceleration in closing kinetics observed for this and the IVS4C chimera, instead the results suggest that the substitution of these voltage sensors in Ca_V_3.3 with those of Ca_V_1.2 channels speeds tail currents primarily by affecting channel gating.

These results indicate that the effects of S4 voltage sensors are not limited to determining the low-voltage dependence of activation of Ca_V_3.3 channels; on the contrary they also contribute to additional properties of LVA behavior as observed for the steady-state inactivation, recovery and deactivation kinetics of the channel.

### The severely reduced current density in IIS4C chimera is mainly due to a mechanistic effect

Considering its fundamental contribution for the low-voltage activation behavior, we further investigated the IIS4C chimera. As shown in Fig 2 and Table 1, this chimera either alone or in double chimeras with IS4C or IVS4C voltage sensors, generates very tiny inward currents such that current density was at least 30-fold smaller than that of Ca_V_3.3 channels. However, we observed that outward currents were not affected in the same magnitude. For instance, the average current density at +80 mV was 39.1 ± 4.1 pA/pF (*n* = 37) for the wild-type channel and 12.0 ± 1.3 pA/pF (*n* = 21) for the IIS4C chimera. In other words, maximal inward current was reduced more than 30-times but outward current at +80 mV was diminished only by a factor of 3. Even more, at +150 mV the respective values were 314.6 ± 41.6 pA/pF (*n* = 11) and 152.5 ± 14.8 pA/pF (*n* = 19), meaning that the more positive the depolarization the more IIS4C channels activate. The same tendency was observed with outward currents evoked by the tail currents protocol used to generate the activation curves of Ca_V_3.3-WT and IIS4C chimeric channels of S1 Fig. A possible explanation for this data is that the IIS4C channel might be requiring more energy (very positive potentials) to fully activate in comparison with the wild-type channel, which is also supported by the lowered voltage dependence for the activation of these channels suggested by the increase in the slope values of the activation *I-V* curves (Table 1). This possibility would assume that protein expression of IIS4C chimera was not significantly affected for the interchange of the voltage sensor of Domain II, but rather that the smaller inward current could be due to an effect on the activation mechanism involving the requirement of a higher energy to activate the channel. We performed two additional sets of experiments in order to obtain more evidences supporting this interpretation. First, by GFP-tagging the chimeras and the wild-type Ca_V_3.3 channel we looked for cell localization of the channels in HEK-293 transfected cells. Confocal images confirmed that the expression of chimera IIS4C at the plasma membrane, and also at intracellular membranes, was quite similar to that observed for the wild-type channel (S2 Fig, panel A). Furthermore, western blot analysis of total protein in a similar stock of transfected cells showed similar amounts of protein for Ca_V_3.3 wild-type, and chimeras IIS4C, and IVS4C (S2 Fig, panels B and C). In addition, two more evidences indicate that IIS4C robust outward currents maintain the LVA properties related to pharmacological and holding potential parameters. Thus, currents of Ca_V_3.3 and IIS4C channels showed almost the same sensitivity to the block by 10 μM TTA-A2 [55] and 5 μM mibefradil (S3 Fig); and to the changing in holding potential from -100 to -60 mV (S4 Fig). In other words, swapping of IIS4 voltage sensor from Ca_V_1.2 to Ca_V_3.3 channel transfers the property of high-voltage activation to the LVA channel, but do not modify its sensitivity to the holding potential or to the blocking by mibefradil and TTA-A2. Altogether, these results suggest that the severe reduction in current density observed in chimera IIS4C might be mainly due to a mechanistic effect that makes the channel less voltage dependent requiring very positive potentials to fully activate (most likely a reduction in open channel probability), rather than an issue with the protein channel expression. Other possibilities, although less unlikely, could be an effect on the unitary conductance of the IIS4C chimeric channels or an induced rectification of the channel.

### Effects of the complementary chimera IIS4I on the gating of Ca_V_1.2 channels

**Fig 7:**
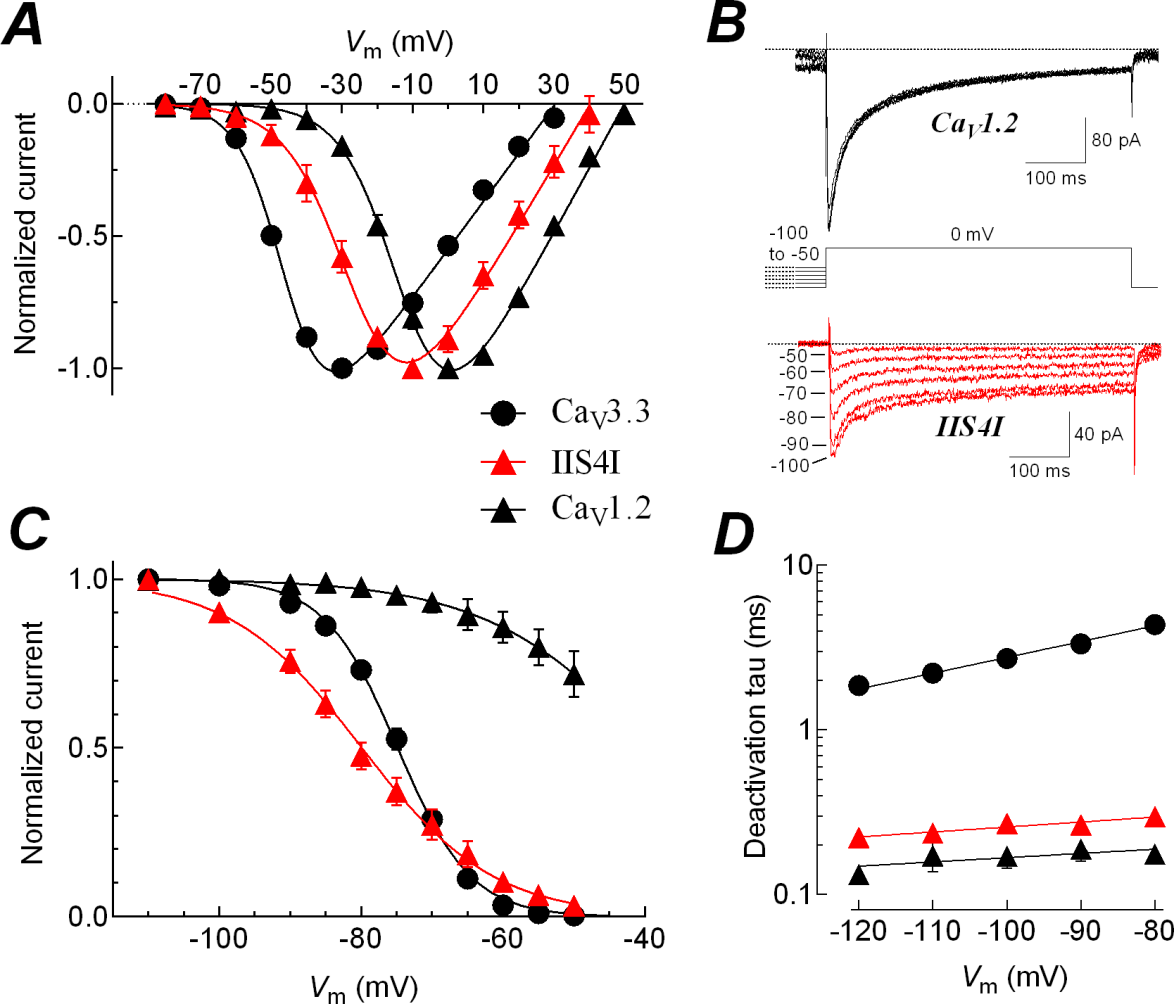
The complementary chimera IIS4I shifts the gating of Ca_V_1.2 channels towards low-voltage behavior. ***A***, normalized *I-V* curves for Ca_V_3.3, IIS4I, and Ca_V_1.2 channels. For clarity purposes only inward currents are plotted. Solid lines are the best fits to the data with a modified Boltzmann function (see Material and Methods). Data in graphs represent mean ± SEM. Parameter values, number of cells and statistical significance are shown in Table 1. ***B***, representative currents recorded at 0 mV from Ca_V_1.2 (black traces) or IIS4I chimeric (red traces) channels. Current recordings were obtained after 15-s prepulses to potentials between -100 and -50 mV, as shown by the voltage protocol. For clarity purposes only the last 50 ms of the prepulses are shown. Note that chimeric channels availability decreases proportionally as the *V*_m_ become more positive, similar to the behavior of Ca_V_3.3-WT channels (see Fig 4). ***C***, steady-state inactivation curves for Ca_V_3.3, IIS4I, and Ca_V_1.2 channels. Currents at 0 mV like those shown in *B* (or at -30 mV for Ca_V_3.3) were normalized to the value at -100 (-110 for Ca_V_3.3) mV, averaged and plotted as a function of the prepulse potential for each channel. Smooth lines are fits to Boltzmann functions. It should be noted that fitting of Ca_V_1.2 data was performed by constraining the Boltzmann function to go from 1 to 0 (as for all the other channels listed in Table 2), although the experimental data reach only a value of 0.72 at -50 mV. In other words, the obtained *V*_1/2_ value is in fact an extrapolation of the fitted data. Data in graphs represent mean ± SEM. Parameter values, number of cells and statistical significance are shown in Table 2. ***D***, time constants of channel deactivation for Ca_V_3.3, IIS4I, and Ca_V_1.2 channels. Time constants were estimated by fitting tail currents to a single exponential function and plotted as a function of the repolarizing potential. Time constants at -90 mV were: 3.33 ± 0.15 ms for Ca_V_3.3-WT (*n* = 25); 0.27 ± 0.04 ms for IIS4I (*n* = 8); and 0.19 ± 0.03 ms for Ca_V_1.2-WT (*n* = 7). Data in graphs represent mean ± SEM. Smooth lines are single exponential fits to the averaged tau values.

In order to investigate whether the voltage sensor of Domain II of Ca_V_3.3 was able to modify the HVA behavior of Ca_V_1.2 channels, we constructed the chimera IIS4I, by swapping the IIS4 segment of Ca_V_3.3 into Ca_V_1.2 channels. The chimera generated currents that were similar in amplitude to those of the Ca_V_1.2-WT channel (current density was -10.4 ± 2.3 for the chimera and -11.3 ± 2.7 pA/pF for the wild-type channel; *n* = 16 cells for each channel; Table 1). The voltage-dependence of activation for the chimera was shifted about 15 mV to more negative potentials, as compared with the Ca_V_1.2-WT channel curve (Fig 7A, Table 1), in other words, the influence of the individual IIS4 voltage sensor of Ca_V_3.3 channel resulted in a LVA-like gating of Ca_V_1.2 channels. This shift was not accompanied by significant changes in the slope of the current-voltage relationship. In concordance with the effects observed for the chimeras IIS4C, the *V*_rev_ value was significantly more negative than that obtained for Ca_V_1.2-WT channel (Table 1). On the other hand, the availability of the channels to be activated after a long depolarizing prepulse was also strongly affected in the chimera IIS4I when compared with the HVA channel. As shown in Fig 7B, current amplitude at 0 mV diminished progressively as the prepulse potential become more positive for chimera IIS4I (red traces), whereas the effect on the current amplitude of the Ca_V_1.2-WT channel was rather discrete (black traces). Therefore, the steady-state inactivation curve of this chimera was severely shifted to more negative potentials when compared with Ca_V_1.2-WT channel (Fig 7C). In fact the *V*_1/2_ for the inactivation of chimeric IIS4I channels was almost 5 mV more negative than the Ca_V_3.3 channels themselves (Table 2). In addition, the Boltzmann constant (*k*) of the chimeric channel inactivation curve showed a discrete tendency towards the Ca_V_3.3 channels values (Table 2). The current kinetics of the chimeric channel were similar to the Ca_V_1.2-WT channel, and even though deactivation of chimeric channels was around 50% slower than the Ca_V_1.2 channel, this difference was minimal when compared with the LVA channel, i.e., Ca_V_3.3-WT (Fig 7D). Thus, the IIS4 segment of Ca_V_3.3 channel translates some LVA properties to the HVA Ca_V_1.2 channel, mainly for the voltage dependence of gating.

### Contribution of the S4-S5 linker of Domain II in the LVA behavior of Ca_V_3.3 channels

**Fig 8:**
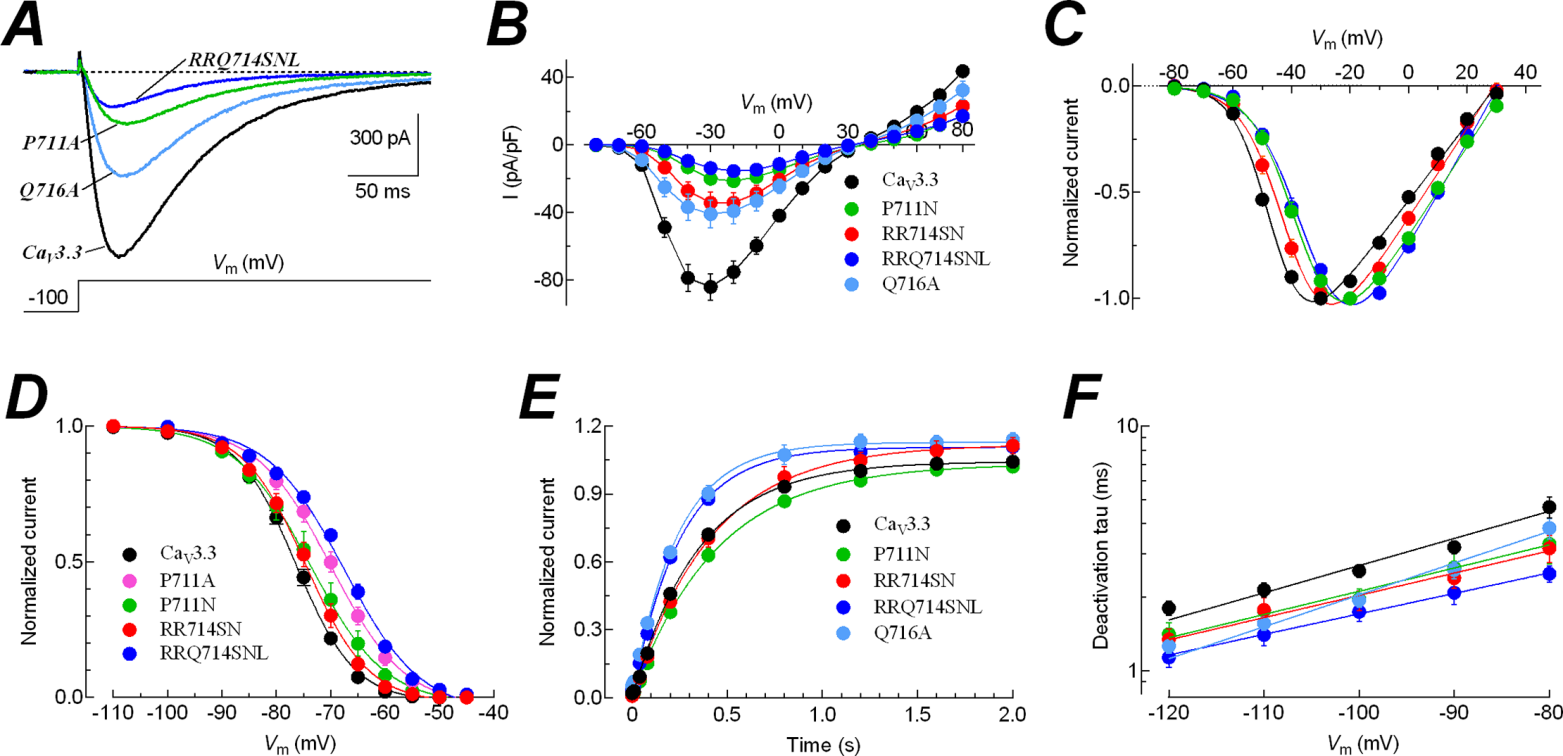
Mutagenesis in Domain II reveals a participation of the S4-S5 linker in the LVA behavior of Ca_V_3.3 channels. ***A***, examples of calcium currents generated by the indicated channels. Holding potential was -100 mV, and current recordings were obtained at -30 mV for Ca_V_3.3-WT and Q716A, and at -20 mV for P711A and RRQ714SNL. ***B***, *I-V* relationships. Current density was calculated for each cell, averaged, and plotted as a function of test potential. ***C***, normalized *I-V* curves for inward currents. Data were fitted with a modified Boltzmann function (continuous line). Same cells as shown in ***B***. ***D***, steady-state inactivation curves for WT and the indicated Ca_V_3.3 mutants. Smooth lines are fits to Boltzmann functions. ***E***, time course of recovery from inactivation at -100 mV for the indicated Ca_V_3.3 channels. Data was analyzed and plotted as indicated in Fig 5. ***F***, deactivation time constants for channel closing as a function of the repolarizing potential. Data in graphs represent mean ± SEM. Parameter and tau values, number of cells and statistical significance are shown in Table 3.

Because IIS4C chimera showed the most significant effects in determining the LVA behavior of Ca_V_3.3 channels, and considering that the segment swapped in the construction of such chimera included the sequence ALRRQL (Fig 1), which has been located in the S4-S5 linker region of Domain II by recent structural data of Ca_V_1.1 channel [42], we further investigate the contribution of some of these residues by introducing single, double and triple mutations. Both channels, Ca_V_1.2 and Ca_V_3.3, share exactly the same positively charged residues from R2 to R6 (Fig 1), however the latter has two additional arginines (R714 and R715) and a proline (P711) in the intracellular-half of the IIS4 segment. Due to the participation of these residues in the activation of voltage-gated ion channels [56,57], we changed them for alanines and for the corresponding residues from the Ca_V_1.2 channel. The results from the electrophysiological characterization of such mutants are summarized in Fig 8 and Table 3. While all the mutants displayed current densities significantly smaller than the wild type channel (Table 3), none of them generated currents as small as those observed for IIS4C chimera (Table 1). Interestingly, single mutants of proline 711 (P711A and P711N) lead to Ca_V_3.3 channels requiring more positive voltages for activation (12 and 10 mV, respectively; Table 3); although the substitution for alanine (P711A) showed a more robust HVA behavior for the steady-state of inactivation than when proline was substituted for the asparagine (P711N) from Ca_V_1.2 channel (Fig 8D). However, the substitution of proline for alanine or asparagine did not significantly modify the recovery from inactivation of the Ca_V_3.3 channel (Fig 8E; Table 3).

**Table 3:** Biophysical parameters of Domain II mutants of Ca_V_3.3.

Channel	Density	Activation			Inactivation			Kinetics			
	pA/pF	*V*_1/2_ (mV)	*k* (mV)	*n*	*V*_1/2_ (mV)	*k* (mV)	*n*	τ_deact_ (ms)	*n*	τ_rec_ (ms)	*n*
Ca_V_3.3-WT	-84.3 ± 7.9	-46.6 ± 0.7	5.5 ± 0.2	40	-76.4 ± 0.6	4.9 ± 0.1	31	3.2 ± 0.2	19	381 ± 25	27
P711A	-17.3 ± 3.3^*a*^	-34.5 ± 1.2^*a*^	7.7 ± 0.3^*a*^	12	-69.4 ± 1.3^*a*^	6.8 ± 0.4^*a*^	10	2.2 ± 0.1^*a*^	10	397 ± 33	9
P711N	-21.5 ± 3.2^*a*^	-36.8 ± 1.4^*a*^	7.1 ± 0.3^*a*^	13	-74.0 ± 1.7	6.1 ± 0.1	9	2.6 ± 0.4	9	454 ± 32	10
RR714AA	-21.5 ± 2.7^*a*^	-41.8 ± 1.1^*a*^	6.4 ± 0.2^*a*^	25	-76.7 ± 0.8	5.9 ± 0.5	15	2.1 ± 0.2^*a*^	12	455 ± 24	14
RR714SN	-34.3 ± 5.6^*a*^	-40.8 ± 1.4^*a*^	6.7 ± 0.3^*a*^	15	-74.7 ± 1.0	5.5 ± 0.2	14	2.4 ± 0.2	10	422 ± 25	9
RRQ714SNL	-15.7 ± 2.3^*a*^	-33.3 ± 1.4^*a*^	8.2 ± 0.2^*a*^	15	-67.3 ± 1.3^*a*^	7.5 ± 0.7^*a*^	14	2.1 ± 0.2^*a*^	13	257 ± 17^*a*^	12
Q716A	-41.2 ± 8.1^*a*^	-44.3 ± 1.6	7.6 ± 0.3^*a*^	14	-75.7 ± 0.7	5.7 ± 0.3	15	2.6 ± 0.2	16	244 ± 12^*a*^	14

Values are given as mean ± SEM. Current density, activation and inactivation parameters were obtained as described in Tables 1 and 2. Deactivation time constants (τ_deact_) at -90 mV were estimated by fitting tail currents to a single exponential function and τ_rec_ was obtained from single exponential fits to the recovery from inactivation data at -100 mV. The number of investigated cells is shown in *n* columns.

^*a*^ Statistical significance when using analysis of variance (*P* < 0.01) followed by Dunnett´s multiple comparison test against Ca_V_3.3-WT.

The contribution of the two arginines to the HVA behavior was more discrete and limited to the voltage-dependence of activation. When mutated for alanines (RR714AA) or the corresponding residues from Ca_V_1.2 channel (RR714SN) the activation curve was significantly shifted to more depolarized potentials by about 5 and 6 mV, respectively (*P* < 0.01; Table 3). Something similar was observed with the glutamine 716 mutant (Q716A), whose electrophysiological parameters were not different from the wild type channel, except that the recovery from inactivation was faster (Table 3). The observed effects with these double and single mutant were boosted with the triple mutation (RRQ714SNL), where the voltage dependence of activation was 13 mV more positive than the WT channel (Fig 8C), while the steady-state inactivation curve was shifted 9 mV in the same direction (Fig 8D). Also the closing of the channel and the time course of recovery from inactivation were faster for this triple mutant compared with the WT channel (Table 3). Together, these results indicate that amino acids like P711 and the tripeptide RRQ (714–716) have an important contribution to the low-voltage activation of Ca_V_3.3 channel. In fact, the results obtained with the mutant RRQ714SNL (~13 mV shift in the *V*_1/2_ of activation; Table 3) and those with the IIS4C chimera (~37 mV shift in the *V*_1/2_ of activation; Table 1), indicate that most of such 37 mV (around 2/3) were due to the contribution of the actual IIS4 segment sequence, while around only 1/3 could be associated with the residues of the S4-S5 linker (Fig 1). In other words, the differences in IIS4 sequences between Ca_V_3.3 and Ca_V_1.2 have a preponderant role in channel gating with an important contribution from the S4-S5 linker of Domain II.

## Discussion

### The IIS4 voltage sensor has a major role in the low-voltage activation of Ca_V_3.3 channels

In this study we have demonstrated the relevance of IIS4 voltage sensor to the low-voltage activation behavior of Ca_V_3.3 T-type calcium channel, although IVS4 segment also has significant effects on the low-voltage activation and the fast-deactivation of the Ca_V_3.3 channel. In contrast, the voltage sensors of segments IS4 and IIIS4 showed minor contribution to the low-voltage activation of Ca_V_3.3 channels; interestingly, the IS4 segment seems to play an important role in preventing the inactivation of Ca_V_3.3 channels at extreme hyperpolarized potentials. These results suggest that voltage sensors contribute differentially to the activation process of Ca_V_3.3 channels.

Previous studies in Ca_V_3.1 channels have shown a similar contribution of S4 segments to channel activation [29,30], however, our results show three major differences with such reports. First, the effect of the replacement of IS4 voltage sensor in Ca_V_3.1 with that of Ca_V_1.2 did not produce any significant effect on the voltage-dependence of activation of the T-type calcium channel (Fig 3 from [29]), whereas our corresponding IS4C chimera, in which the IS4 voltage sensor of Ca_V_3.3 was replaced with that of Ca_V_1.2, displayed a significant shift in the *I-V* curve to more negative potentials (almost 9 mV, Table 1), and an even greater shift in the steady-state inactivation curve (19 mV, Table 1). Interestingly, Ca_V_3.1 and Ca_V_3.2 differ in IS4 sequence, having an arginine in RAIN rather than a lysine in Ca_V_3.3’s KAIN (Fig 1). If such a difference in the amino acid sequence accounts for the distinct effects on the voltage sensing of activation between Ca_V_3.1 and Ca_V_3.3 channels, it remains to be elucidated. Second, swapping the IIIS4 segment in the Ca_V_3.1 channel caused a shift of the *I-V* curve towards more positive potentials (around 8 mV) (Fig 5 from [29]). In contrast our equivalent chimera with the Ca_V_3.3 channel showed a small shift to more hyperpolarized potentials (Table 1). And third, the swapping of IIS4 voltage sensor in Ca_V_3.1 with the corresponding region from Ca_V_1.2 channels produced the most drastic effect in the voltage dependence of activation of Ca_V_3.1 channels towards more positive potentials (about 20 mV), but still far from the Ca_V_1.2 channel *V*_1/2_ of activation by around 25 mV (Fig 4 from [29]). In the present study, the swapping of the IIS4 voltage sensor between Ca_V_3.3 and Ca_V_1.2 channels (chimera IIS4C) produced channels with an *I-V* curve that almost overlaps with that of the Ca_V_1.2 channel (Fig 2C), i.e., the *V*_1/2_ for IIS4C chimeric channels was shifted almost 40 mV to more positive potentials in comparison with Ca_V_3.3-WT channels (Table 1). A likely explanation for this difference in the magnitude of the *V*_1/2_-shifting could be associated to the differences in the sequence of segments swapped. The segment substituted in the present work of Ca_V_3.3 has an extra arginine compared with the one of Ca_V_3.1 (Fig 1); and is longer by two residues (Q and L) at the intracellular end compared with that swapped in Ca_V_3.1 [29]. In fact, our results show a significant role of this region of Ca_V_3.3 as the mutant RRQ714SNL shifted the *V*_1/2_ of activation by 13 mV to more positive potentials compared with the Ca_V_3.3-WT channel (Table 3). According to the structural data obtained recently by cryo-electron microscopy from the rabbit Ca_V_1.1 channel [42], the triplet RRQ forms part of the S4-S5 linker region of Domain II. Thus, these results indicate that the whole effect observed with the IIS4C chimera in the low-voltage activation behavior of the Ca_V_3.3 channel would be the sum of the contribution of the IIS4 segment and the triplet RRQ from the S4-S5 linker of Domain II.

The evidence provided by the Wray group [29,30], strongly suggest a contribution of additional structural elements to the S4 segments in each Domain of α1 subunit that regulates the low-voltage gating of Ca_V_3.1 channels. In fact they found that, except for Domain II, all other Domains (I, III and IV) shifted the *I-V* relationships of Ca_V_3.1 channels towards more depolarized potentials, very close to the *I-V* curve for the Ca_V_1.2 channels. However, they also reported that just by swapping the S4 voltage sensor of this domain between Ca_V_3.1 and Ca_V_1.2 channels, the resulting chimera showed an *I-V* curve that was shifted about 20 mV to more positive potentials, suggesting the crucial role of S4 voltage sensor of Domain II for Ca_V_3 channels. In the present study, we found that IIS4 voltage sensor, with an important contribution of some residues from the S4-S5 linker, has the most dominant effect, followed by the IVS4 segment, in the low-voltage activation behavior of Ca_V_3.3 channels. In this regard, a more recent study has shown that the activation of Ca_V_3.2 channels might be the result of cooperative contribution of residues from the S4-S5 linker of Domain II and the S6 helices from Domains II and III [58]. In such a scenario, the results presented here for Ca_V_3.3 channel might be due to the disruption of structural interactions between S4 voltage sensors, S4-S5 linkers, and S6 helices, that leads to the gating of the channel and, therefore, it can be anticipated that such interactions could be more relevant in the case of Domain II of Ca_V_3.3 channels. In this context, our results and those reported for Ca_V_3.1 channels [29,30], suggest a general pattern for T-type calcium channels activation, although subtle differences, as those shown here for Ca_V_3.3 channels, might be expected when studying each individual channel. It remains to be investigated if this is the case for Ca_V_3.2 channels. As shown recently, the differences in gating among T-type calcium channels are present even at the isoform level of channels [33,59].

It is worthy of note that the IVS4 voltage sensor also has an important role on such behavior as its substitution for that of the Ca_V_1.2 channel shifted the *V*_1/2_ of activation by 16 mV to more depolarized potentials (Table 1). Some support for the role of Domain IV voltage sensor in activation sensing was shown in Ca_V_3.1 channels [29], and also from Zamponi´s group who studied the gating properties of Ca_V_3.1 and Ca_V_3.3 channels by constructing chimeras between these two LVA channels [41]. They described a role for Domain IV in the Ca_V_3.3 channel gating, however, this effect (around 6 mV shift in the *I-V* towards more negative potentials) was observed in a chimera where Domain IV and the carboxy terminus of Ca_V_3.3 channel was substituted by that of a Ca_V_3.1 channel. Additional evidence for the role of IVS4 voltage sensor in Ca_V_3 channels gating came from a study focused on the neutralization of the three outermost arginines (R) in Domain IV of Ca_V_3.2 channels [60]. The research showed that the neutralization of R2 and R3 with glutamine or cysteine residues led to a significant positive shift of Ca_V_3.2 channels *I-V* curve, though R1 (the most external arginine) did not have significant effects on any of the channel biophysical properties. Finally, we cannot rule out the possibility that the effects on channel gating observed with chimera IVS4C might involve the participation of the S4-S5 linker residues included in this chimera (Fig 1). Further mutagenesis experiments are required to address this issue.

### Influence of IIS4 voltage sensor on additional LVA properties of Ca_V_3.3 channels

In addition to the voltage dependence of activation, in this study we investigated the influence of S4 voltage sensors in other biophysical parameters of the low-voltage activation behavior of Ca_V_3.3 channels. Particularly, the closing of the channels, the recovery and the voltage dependence of steady-state inactivation. Channel closing kinetics from the open state (deactivation) is a common characteristic used to discriminate between LVA and HVA channels. We found that IIS4 voltage sensor is also crucial for the slow-deactivating characteristic of Ca_V_3.3 channels, followed by the IVS4 voltage sensor. Both significantly accelerated the closing kinetics of the channel, whereas the IS4 voltage sensor had a smaller effect and the IIIS4 had no influence at all on the deactivation of the Ca_V_3.3 channel (Fig 6B). Therefore, it can be concluded that IIS4 and IVS4 voltage sensors of Ca_V_3.3 channel contributes to the stabilization of the channel in the open state, preventing a faster transition from this to the closed state. So far, there is no data on the literature to directly compare our results with, as the existing studies concentrated only on the voltage-dependence of activation, and activation and inactivation kinetics [29,30]. However, the study of Hanck’s group [60] reported a positive shift of Ca_V_3.2 channels *I-V* curve after the neutralization of R2 and R3 arginines of IVS4 voltage sensor with glutamine or cysteine residues, which was accompanied by an acceleration of the tail current kinetics. On the contrary, the deactivation time constant of Ca_V_3.1 channels was significantly slowed down as a result of neutralization of the outermost arginine in IVS4, and also in the IIIS4 segments [32].

Interestingly, the substitution of S4 segments in Ca_V_3.3 for those of Ca_V_1.2 channel led to very modest changes, if any, in the steady-state inactivation curves of most chimeras. The exceptions were IIS4C chimera, which caused a small but significant positive shift (around 8 mV), and the IS4C chimera that promoted 19 mV shifts in the inactivation curve to more hyperpolarized potentials with respect to the Ca_V_3.3 channel, either with the single substitution of Domain I voltage sensor (chimera IS4C) or when it was in combination with the voltage sensor of Domain III (double chimera IS4C-IIIS4C; Table 2). The importance of IS4 voltage sensor for the voltage dependence of inactivation was better appreciated with the double chimera IS4C-IIS4C, where the *V*_1/2_ value was -83.3 ± 2.2 mV, compared with the -68.4 ± 1.7 mV displayed by the IIS4C single chimera, which in turn had a significant small shift to more positive potentials when compared with Ca_V_3.3 channels (Table 2). Interestingly, the extremely strong effect of IS4C chimera on the inactivation curve was somehow unexpected. Previously, we have shown a similar effect on the steady-state inactivation by disrupting the structure of a particle termed the “gating brake” that resides in the amino end of the I-II loop in Ca_V_3 channels [44,47,61]. Unfortunately, previous similar studies in Ca_V_3.1 channels did not report effects about the voltage dependence of steady-state inactivation of the constructed chimeras [29,30], thus it is difficult to suggest whether the effect of IS4 voltage sensor on steady-state inactivation present here for Ca_V_3.3 channels is a conserved characteristic among the Ca_V_3 channels subfamily. In this regard, substitution of the whole Domain I of Ca_V_3.3 channel for that of Ca_V_3.1 channel did not have any effect on the voltage dependence of inactivation of the channels, and the effect the authors found with the equivalent swapping of Domain III was in the opposite direction, making the Ca_V_3.3 channel to inactivate at more positive potentials [41]. In another study where the outermost arginines in the Ca_V_3.1 channels were neutralized with cysteines, the authors reported that the four individual neutralizations shifted significantly the quasi steady-state inactivation of the channels to more negative potentials (from 7 to 17 mV) compared with the Ca_V_3.1 channel; with the neutralization of the arginine of Domain III voltage sensor (R1379C) inducing the most drastic effect [32]. Therefore, the role of the outermost arginines in Ca_V_3 channels might not represent the behavior of the whole voltage sensor, at least in the voltage dependence of inactivation of the channels.

On the other hand, it has been shown in Ca_V_3.2 channels that single and double mutations of residues in IIS4-S5 linker and IIIS6 helix led to a 25 mV negative shift in the steady-state inactivation curve [58]. Therefore, one possible explanation of our results in Ca_V_3.3 channels could be that IS4 segment, the IS4-S5 linker or even the IS6 segment, have structural interactions with the “gating brake” structure localized in the I-II loop, and that the substitution of this voltage sensor with that of Ca_V_1.2 does not restore such interactions leading the channels to inactive from closed states at very negative potentials. In fact, a 3-D model of the gating brake predicts that the second helix of the gating brake establishes important contacts with the gating machinery, including the IS4-S5 linker, thereby stabilizing a closed state of T-type calcium channels [62].

### On the voltage sensing mechanism of Ca_V_ channels: Relations between functional and structural data

Recently, the structure of the Ca_V_1.1 channel was resolved by cryo-electron microscopy at 3.6 Å resolution [42]. First of all, the structure of this HVA channel shows that our original design of chimeras included 5 to 8 more residues at the intracellular end than the actual S4 segments found in the Ca_V_1.1 structure. Those extra amino acids would be forming part of the S4-S5 linker, which has been already described as an important motif for the gating of Ca_V_3.2 channels [58], but its contribution to the differences in gating threshold between LVA and HVA channels has not been addressed. Regarding the meaning of our data about the crucial role of IIS4 and IVS4 voltage sensors in the low-voltage activation of Ca_V_3.3 channels under the light of the Ca_V_1.1 channel structure some considerations could be formulated. A shared characteristic between VSD_II_ and VSD_IV_ is that they include the negative residue on transmembrane helix S3 (aspartic acid) that is part of the charge transfer center (CTC) [63], while this residue is not present in the other two VSDs (S5 Fig). Structural and functional data obtained from voltage-gated ion channels (potassium and sodium mainly) have shown that this Asp residue on S3, together with two additional residues in S2, one negatively charged (Glu) and other non-polar residue (Phe), form the CTC, which supports the voltage-dependent transition from the resting to the activated state of the S4 positively charged residues [63–65]. Therefore, because the sequence of HVA and LVA channels is identical in such residues (D673 in S3 of VSD_II_ and D1520 in S3 of VSD_IV_ of the Ca_V_3.3 used here), we speculate that this difference between VSD_II_ and VSD_IV_ with VSD_I_ and VSD_III_ could be a partial explanation of our data showing the crucial contribution of these two voltage sensors in the proper gating of Ca_V_3.3 channels, and in general for the Ca_V_ channels family. However, such explanation does not account for the difference in gating between LVA and HVA channels, as the swapping of IIS4 and IVS4 voltage sensors from Ca_V_1.2 to Ca_V_3.3 channels does not include the Asp residue in segment S3, and this is present in both channels. More recently, it has been shown the participation of an additional Asp residue on S3 (named D4), outside the CTC of the fourth VSD of Ca_V_1.1 channel (S5 Fig) that is regulated by the alternative splicing of the S3-S4 linker [66]. The authors found that the Ca_V_1.1 isoform with the shorter S3-S4 linker (Ca_V_1.1e) generates 6-fold larger currents and ~26 mV left shift in voltage-dependence compared with the longer S3-S4 linker isoform (Ca_V_1.1a), and that the neutralization of this aspartate by mutating it to asparagine reverted the Ca_V_1.1e current properties to those of Ca_V_1.1a. Interestingly, the D4 residue is conserved in 6 out of 7 HVA channels, but in LVA channels the position is occupied by a glycine (S5 Fig), and the corresponding S3-S4 linker is also as short as the one of Ca_V_1.1e. The pro-HVA role of D4 in our results with the S4 voltage sensors chimeras was not observed, because even in its absence the chimera IVS4C shifted the gating properties of Ca_V_3.3 channels towards those of Ca_V_1.2. Therefore, despite the demonstrated participation of additional structures to the S4 segment itself (like S3-S4 and S4-S5 linkers) and individual amino acids (like those of CTC and D4) in the mechanism for the proper voltage-sensing of Ca_V_ channels, the molecular substrate for the differences in gating between LVA and HVA remains elusive. Our results demonstrate that swapping the S4 voltage sensors of Domain II and IV, together with some residues from the S4-S5 linkers, is enough to induce an HVA-like gating behavior of Ca_V_3.3 channels, and in concordance with the data from other groups discussed here, suggest the necessity to perform additional studies to dissect the contribution of each residue in the S4 voltage sensors (not only the positively charged residues but also those that are around them), to obtain more insights about the differences in gating between LVA and HVA channels.

In conclusion, the results of the chimeric analysis of the S4 voltage sensors presented here revealed the crucial role of S4 voltage sensor and the S4-S5 linker of Domain II in the LVA behavior of Ca_V_3.3 channels, followed in importance by the Domain IV voltage sensor. On the contrary, voltage sensor of Domain I and III did not show significant relevance for the low-voltage activation characteristic of Ca_V_3.3 channels, although the IS4 voltage sensor alone had a strong influence in preventing the inactivation of Ca_V_3.3 channels from closed-states. It seems to be that the outstanding contribution of the IIS4 and IVS4 voltage sensors for the LVA properties of Ca_V_3.3 channel is concerted through the residues of the S4 voltage sensors themselves, and also by structural interactions with other regions of the channel.

## Supporting information

S1 Fig Voltage-dependence of activation for CaV3.3 and IIS4C chimera determined with an instantaneous *I-V* protocol. ***A***. Representative family currents recorded from HEK-293 cells transfected with Ca_V_3.3 (black traces) or IIS4C chimeric (red traces) channels by using the indicated voltage protocol. Twenty-ms depolarizing pulses were applied from -80 to +120 mV in 10-mV steps from a HP of -100 mV. Tail currents were recorded at -120 mV in order to promote larger currents in the IIS4C chimera. The characteristics of each channel are observed, significantly larger inward currents and slower tail currents for the wildtype channel in comparison with the chimeric channel. ***B***. Activation curves. Tail current amplitudes were normalized to the maximum value and plotted as the fraction of open channels against test potential. Data points were fit with a Boltzmann function of the form: *I* = *I*_max_ */* (1 + exp ((− *V*_m_ − *V*_1/2_)*/k*)). The obtained parameters *V*_1/2_ and *k* were: -11.1 ± 3.4 mV and 17.4 ± 0.9 mV (n = 7) for Ca_V_3.3; and +45.9 ± 4.0 mV and 19.2 ± 0.6 mV for the IIS4C chimeric channels (n = 6).
(TIF)

S2 Fig The small current density of chimera IIS4C is not associated with a significant reduction of channel expression. ***A*,** localization of wild-type and IIS4C chimeric Ca_V_3.3 channels within HEK-293 cells. Representative confocal images showing subcellular distribution of Ca_V_3.3 and IIS4C chimera in HEK-293 cells after 48 h of transfection. Both channels were GFP-tagged (green), and cell membranes were stained with FM4-64 (red). The merged images are shown at the bottom, GFP-tagged channels located in the plasma membrane are seen as punctuate yellow clusters. *Scale bars*, 3.3 μm. Representative data from 12–15 cells from three independent experiments. ***B***, expression of Ca_V_3.3-WT and IIS4C chimera in HEK-293 cells. Western blot of protein homogenates from untransfected HEK-293 cells (HEK-293), or transiently transfected with Ca_V_3.3-GFP, IIS4C-GFP or IVS4C-GFP. Blots were probed with anti-GFP (top), and β-actin was used as a loading control (bottom). Results are representative of three independent experiments with similar results. ***C***, bar chart showing quantitation of three experiments like the one shown in ***B***. Data in graphs represent mean ± SEM. The data were corrected with the loading control (β-actin). There were no statistical differences among the means.
(TIF)

S3 Fig The pharmacological profile of IIS4C currents agrees with the CaV3.3 channels. Examples of family currents obtained from Ca_V_3.3 and IIS4C chimeric channels before (Control), during the exposure to mibefradil or TTA-A2, and after washing out the corresponding compound (Recovery). Currents were evoked by 10 mV-step depolarizations starting at -80 mV (for Ca_V_3.3) or at -40 mV (for IIS4C) until +120 mV, in both cases, from a HP of -100 mV. Traces in red denotes the peak of the inward current for each channel. The blocking effect of Mibefradil and TTA-A2 was practically the same on wildtype and IIS4C chimeric Ca_V_3.3 channels. Mibefradil: Ca_V_3.3, 89.6 ± 2.4% at -30 mV, and 79.6 ± 4.4% at +120 mV (n = 2); IIS4C: 87.1 ± 7.8% at 0 mV, and 93.5 ± 4.0% at +120 mV (n = 3). TTA-A2: Ca_V_3.3, 79.4 ± 10.2% at -30 mV, and 64.6 ± 14.7% at +120 mV (n = 2); IIS4C: 85.8 ± 7.8% at 0 mV, and 75.3 ± 3.4% at +120 mV (n = 4).
(TIF)

S4 Fig Amplitude of IIS4C currents is holding-potential dependent as LVA channels. Representative current traces at the indicated voltages for Ca_V_3.3, IIS4C and Ca_V_1.2 channels evoked by using a holding potential (HP) of -100 mV (left panels) or -60 mV (right panels). As a typical LVA channel, currents of Ca_V_3.3 are severely reduced in response to the changing the HP from -100 to -60 mV. On the contrary, the Ca_V_1.2 currents are practically unaffected for this experimental maneuver. In agreement with the LVA behavior, currents amplitude of IIS4C chimera was equally dependent on the HP as for Ca_V_3.3 channels. In order to obtained larger currents of IIS4C chimera, for these experiments the concentration of the charge carrier in the external solution was increased from 5 to 10 mM Ca^2+^. In some panels, tail currents mixed with uncompensated capacitive transients are off scale.
(TIF)

S5 Fig Protein sequence alignment from IS2 through IVS4 segments of α1 subunit for human CaV channels. S2, S3 and S4 segments are delineated by black lines above the sequence alignment and amino acids are color-coded: red, positively charged; green, negatively charged; blue, polar; and yellow, hydrophobic. The charge transfer center (CTC) that facilitates the movement of voltage sensors consists of An1 and An2 (negative or polar residues; shaded magenta), and Phe (the occluding residue, shaded cyan) on the S2 segment; and an Asp residue in the S3 segment in each repeat. However, in all Ca_V_ channels this residue is only conserved in VSD_II_ and VSD_IV_ (shaded red), as shown by the alignments. The negative residue near the extracellular end of S3 (outside the charge transfer center of VSD_IV_ that is critical for voltage-sensing of Ca_V_1.1 channels) is highly conserved (shaded green) in all HVA channels, but in LVA channels is missing.
(TIFF)
