# Supplementary figures and images for: Contribution of S4 segments and S4-S5 linkers to the low-voltage activation properties of T-type CaV3.3 channels
Source: PLoS One. 2018 Feb 23;13(2):e0193490. doi: 10.1371/journal.pone.0193490 (PMC5825144; doi:10.1371/journal.pone.0193490)

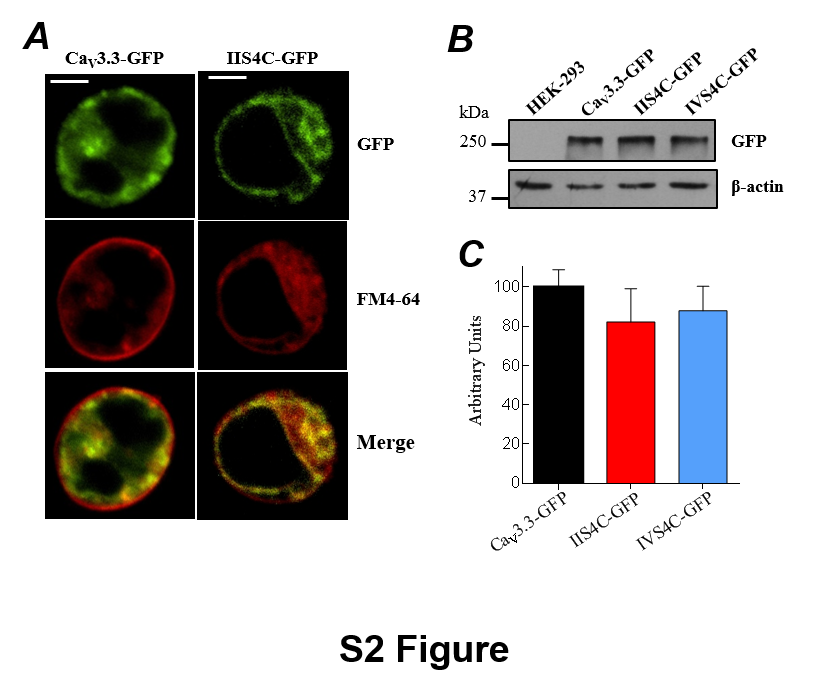

Supplement: S2 Fig — A, localization of wild-type and IIS4C chimeric CaV3.3 channels within HEK-293 cells. Representative confocal images showing subcellular distribution of CaV3.3 and IIS4C chimera in HEK-293 cells after 48 h of transfection. Both channels were GFP-tagged (green), and cell membranes were stained with FM4-64 (red). The merged images are shown at the bottom, GFP-tagged channels located in the plasma membrane are seen as punctuate yellow clusters. Scale bars, 3.3 μm. Representative data from 12–15 cells from three independent experiments. B, expression of CaV3.3-WT and IIS4C chimera in HEK-293 cells. Western blot of protein homogenates from untransfected HEK-293 cells (HEK-293), or transiently transfected with CaV3.3-GFP, IIS4C-GFP or IVS4C-GFP. Blots were probed with anti-GFP (top), and β-actin was used as a loading control (bottom). Results are representative of three independent experiments with similar results. C, bar chart showing quantitation of three experiments like the one shown in B. Data in graphs represent mean ± SEM. The data were corrected with the loading control (β-actin). There were no statistical differences among the means. (TIF) [file pone.0193490.s002.tif]

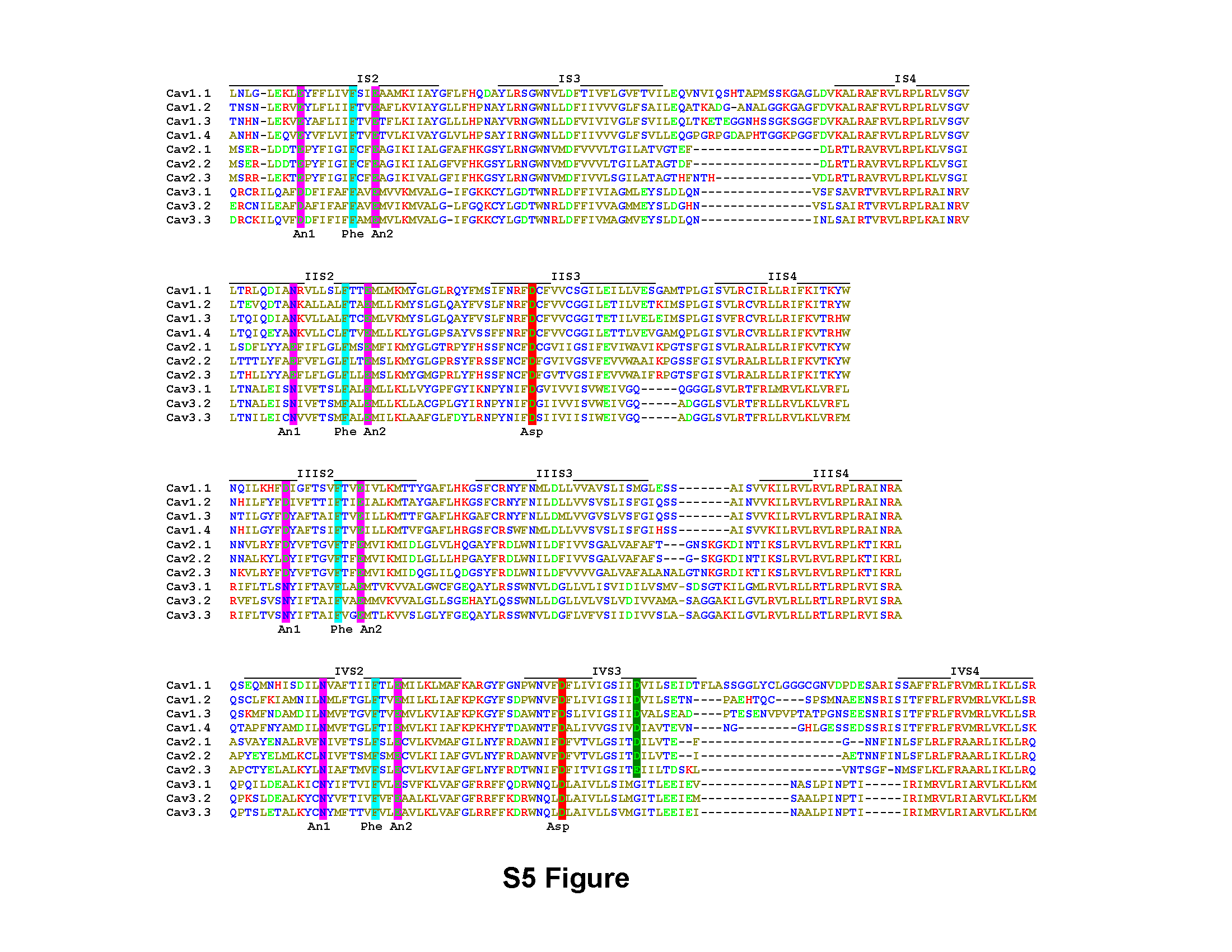

Supplement: S5 Fig — S2, S3 and S4 segments are delineated by black lines above the sequence alignment and amino acids are color-coded: red, positively charged; green, negatively charged; blue, polar; and yellow, hydrophobic. The charge transfer center (CTC) that facilitates the movement of voltage sensors consists of An1 and An2 (negative or polar residues; shaded magenta), and Phe (the occluding residue, shaded cyan) on the S2 segment; and an Asp residue in the S3 segment in each repeat. However, in all CaV channels this residue is only conserved in VSDII and VSDIV (shaded red), as shown by the alignments. The negative residue near the extracellular end of S3 (outside the charge transfer center of VSDIV that is critical for voltage-sensing of CaV1.1 channels) is highly conserved (shaded green) in all HVA channels, but in LVA channels is missing. (TIFF) [file pone.0193490.s005.tiff]
